# The Large Mitochondrial Genome of *Symbiodinium minutum* Reveals Conserved Noncoding Sequences between Dinoflagellates and Apicomplexans

**DOI:** 10.1093/gbe/evv137

**Published:** 2015-07-20

**Authors:** Eiichi Shoguchi, Chuya Shinzato, Kanako Hisata, Nori Satoh, Sutada Mungpakdee

**Affiliations:** Marine Genomics Unit, Okinawa Institute of Science and Technology Graduate University, Onna, Okinawa, Japan

**Keywords:** *Symbiodinium*, *Plasmodium*, mitochondrial genome expansion, RNA editing, gene map, noncoding

## Abstract

Even though mitochondrial genomes, which characterize eukaryotic cells, were first discovered more than 50 years ago, mitochondrial genomics remains an important topic in molecular biology and genome sciences. The Phylum Alveolata comprises three major groups (ciliates, apicomplexans, and dinoflagellates), the mitochondrial genomes of which have diverged widely. Even though the gene content of dinoflagellate mitochondrial genomes is reportedly comparable to that of apicomplexans, the highly fragmented and rearranged genome structures of dinoflagellates have frustrated whole genomic analysis. Consequently, noncoding sequences and gene arrangements of dinoflagellate mitochondrial genomes have not been well characterized. Here we report that the continuous assembled genome (∼326 kb) of the dinoflagellate, *Symbiodinium minutum*, is AT-rich (∼64.3%) and that it contains three protein-coding genes. Based upon in silico analysis, the remaining 99% of the genome comprises transcriptomic noncoding sequences. RNA edited sites and unique, possible start and stop codons clarify conserved regions among dinoflagellates. Our massive transcriptome analysis shows that almost all regions of the genome are transcribed, including 27 possible fragmented ribosomal RNA genes and 12 uncharacterized small RNAs that are similar to mitochondrial RNA genes of the malarial parasite, *Plasmodium falciparum*. Gene map comparisons show that gene order is only slightly conserved between *S. minutu*m and *P. falciparum*. However, small RNAs and intergenic sequences share sequence similarities with *P. falciparum*, suggesting that the function of noncoding sequences has been preserved despite development of very different genome structures.

## Introduction

Mitochondrial (mt) genomes are considered characteristic of eukaryotic cells (Lang et al. 1999; Gray et al. 2004). Although eukaryotic mt genomes are believed to have arisen from alpha-proteobacteria, extant eukaryotes possess either linear or circularized mtDNA with varied and reduced gene content (Lang et al. 1999). For example, most metazoan mt genomes are 13–20 kb, compact, circular molecules, encoding 12–13 proteins, 24–25 transfer RNAs (tRNAs), and 2 ribosomal RNAs (rRNAs). On the other hand, linear mtDNAs with terminal repeats (putative telomeres) have also been found in many species, such as the yeast, *Candida*, and the ciliate, *Tetrahymena* (Lang et al. 1999; Rycovska et al. 2004).

The ciliates, *Paramecium aurelia*, *Tetrahymena pyriformis*, and *Tetrahymena thermophile*, have linear mt genomes of 40–47 kb, which contain approximately 50 genes (Burger et al. 2000; Gray et al. 2004). In contrast, only three protein-coding genes (*cox1* [*cytochrome oxidase subunit I*], *cox3* [*cytochrome oxidase subunit III*], and *cob* [*cytochrome b*]) and fragmented rRNAs (LSU, large subunit; SSU, small subunit) have been identified in mt genomes of apicomplexans and dinoflagellates (Feagin 1992; Nash et al. 2008; Vaidya and Mather 2009). Recent work on the mt genome of the malaria parasite, *Plasmodium falciparum,* found additional fragmented rRNAs and uncharacterized small RNAs (Feagin et al. 2012). Diverse linear mt genomes have been reported in apicomplexans (Vaidya and Mather 2009). For example, *Plasmodium* has mt genomes in tandemly repeated arrays with a unit length of approximately 6 kb. On the other hand, *Babesia* and *Theileria* have monomeric mt genomes (Hikosaka et al. 2012). Previous work on dinoflagellate mt genomes has suggested complex organization, with extensive recombined and fragmented gene copies (Waller and Jackson 2009). Fragmented mt genomes and/or transcripts have been reported in at least 25 dinoflagellate taxa (table 1). The foregoing studies have confirmed that dinoflagellate mtDNA includes *cox1*, *cox3*, *cob* and fragmented rRNAs, and have detailed unusual mRNA characteristics (reviewed by Nash et al. 2008; Waller and Jackson 2009). Extensive RNA editing of the three protein-coding genes (Lin et al. 2002; reviewed in Lin et al. 2008) and *trans*-splicing of *cox3* have been reported (Jackson et al. 2007; Imanian et al. 2012; Jackson and Waller 2013). However, transcripts from the basal dinoflagellates, *Hematodinium* sp. and *Oxyyrhis marina*, did not show RNA editing (Slamovits et al. 2007; Jackson et al. 2012). *Trans*-splicing of *cox3* was not found in *O. marina* (Slamovits et al. 2007). Losses of canonical start and stop codons have also been suggested (Norman and Gray 1997; Jackson et al. 2012; reviewed in Nash et al. 2008). On the other hand, analyses of noncoding sequences have been frustrated by high recombination rates in these genomes (Patron et al. 2005; reviewed in Waller and Jackson 2009). In addition, some reports have suggested that the total dinoflagellate mt genome size is likely to be large (Waller and Jackson 2009; Shoguchi et al. 2013), and the dinoflagellate mt genome is thought to be one of the most complex (Nash et al. 2008). For example, it is estimated that 85% of the mt genome in *Amphidinium carterae* is noncoding (Nash et al. 2007). Although inverted repeat (IR) elements in intergenic regions have been reported, functions of these elements are unknown (Waller and Jackson 2009). Thus, it has been assumed that each alveolate lineage developed different mt genomic structure (Slamovits et al. 2007). Interestingly, recently reported mt genomes of colponemids, an early alveolate lineage, suggest that the ancestral alveolate genome encoded a typical mt gene set (Janouškovec et al. 2013).

**Table 1:** Summary of the Papers Reporting mt Genomes and/or Transcriptomes in Dinoflagellates

Species name	References
*Akashiwo sanguinea*	Zhang et al. (2008)
*Alexandrium catenella*	Kamikawa et al. (2007, 2009)^a^
*Alexandrium tamarense*	Zhang and Lin (2005); Zhang et al. (2008)
*Amphidinium carterae*	Nash et al. (2007)^a^; Jackson and Waller (2013)
*Crypthecodinium cohnii*	Norman and Gray (1997, 2001)^a^; Lin et al. (2002); Jackson et al. (2007)
*Dinophysis acuminata*	Zhang et al. (2008)
*Durinskia baltica*	Imanian and Keeling (2007); Imanian et al. (2012)^a^
*Gonyaulax polyedra*	Chaput et al. (2002)
*Hematodinium* sp.	Jackson et al. (2012)^a^
*Heterocapsa triquetra*	Patron et al. (2005)
*Karenia brevis*	Zhang et al. (2008)
*Karlodinium micrum*	Zhang and Lin (2005); Jackson et al. (2007)^a^
*Kryptoperidinium foliaceum*	Imanian and Keeling (2007); Imanian et al. (2012)^a^
*Oxyrrhis marina*	Slamovits et al. (2007)^a^
*Pfiesteria piscicida*	Lin et al. (2002); Zhang and Lin (2002)
*Pfiesteria shumwayae*	Zhang and Lin (2005)
*Prorocentrum cassubicum*	Zhang et al. (2008)
*Prorocentrum micans*	Zhang and Lin (2005); Zhang et al. (2008)
*Prorocentrum minimum*	Lin et al. (2002)
*Protoceratium reticulatum*	Zhang et al. (2008)
*Pseudopfiesteria shumwayae*	Zhang et al. (2008)
*Scrippsiella* sp.	Zhang et al. (2008)
*Scrippsiella sweeneyae*	Zhang et al. (2008)
*Symbiodinium microadriacticum*	Zhang and Lin (2005); Zhang et al. (2008)
*Symbiodinium* sp.	Zhang and Lin (2005); Zhang et al. (2008); Jackson and Waller (2013)

^a^mt genome sequences were reported.

Our previous work on the endosymbiotic dinoflagellate, *Symbiodinium minutum*, has confirmed the presence of unusual nuclear (Shoguchi et al. 2013) and plastid genomes (Mungpakdee et al. 2014). In addition, this species may possess high mt genome copy numbers (Shoguchi et al. 2013). In this study, by analyzing the wealth of sequence data, we characterized the *Symbiodinium* mt genome and transcriptomes, including many noncoding sequences, and we compared them with mt genomes of *Plasmodium* and dinoflagellates. Assembly of fragmented DNA in general is technically difficult, but physical link information from fosmid end sequencing greatly aided mt genome assembly. Our analysis reveals conserved, noncoding sequences during myzozoan (apicomplexans and dinoflagellates) mt genome evolution. In addition, *Symbiodinium* is a large genus, classified into nine major clades (Coffroth and Santos 2005; Pochon et al. 2014); therefore, the complete *Symbiodinium* mt genome will be an important resource to study populations and environmental adaptations using genomic approaches (Shinzato et al. 2014).

## Results and Discussion

### The De Novo Assembled mt Genome of *S. minutum*

**Fig. 1.—:**
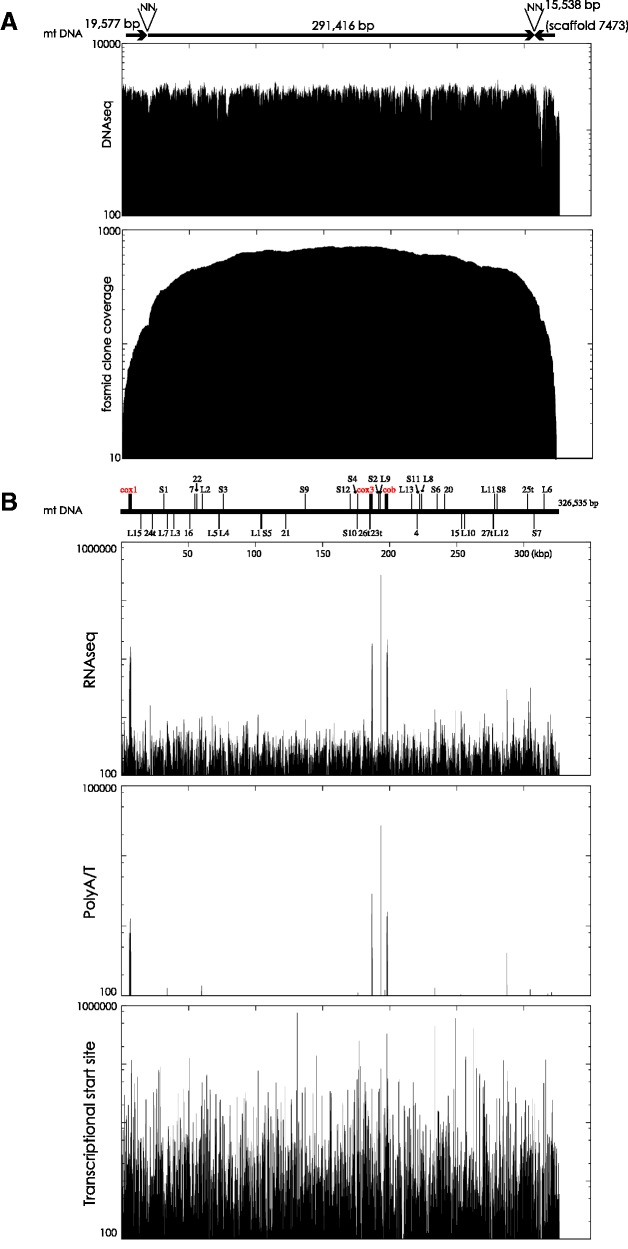
A mitochondrial genome and transcripts in *S. minutum.* (*A*) The assembled mt genome of *S. minutum* showing the high copy number. Arrows show two contigs and one scaffold (scaffold 7473), which are joined by paired-end sequences of fosmid clones and are labeled “NN” because of indeterminate distances. These constitute a scaffold of 326,535 kb. The upper graph indicates the high coverage of illumina reads that were mapped onto the scaffold. The lower plot shows clone coverage by fosmid paired-end mapping, partially supporting the accuracy of the assembly. (*B*) Transcriptomes from mtDNA and possible ends. The *S. minutum* mt genome with predicted genes is shown in upper region. Genes above or below the line indicate the transcription direction. Protein-coding genes are in red. Detailed gene map information is shown in supplementary figure S4, Supplementary Material online, and table 3. The upper graph shows coverage of RNAseq reads from illumina libraries that are enriched RNAs with polyA sequences. High coverage reads are found on *cox1*, *cox3*, fragment E of the ribosomal LSU, and *cob*. Only reads with poly A or T (more than four) are shown on middle graph, suggesting polyadenylated transcripts and potential 3′-ends. The lower graph displays reads from the TSS library, which is enriched RNA with 5′ cap structures, indicating the presence of multiple 5′-ends.

To reconstruct the mt genome of *S. minutum*, 20 analyses using only high coverage illumina paired-end reads (DNAseq) were performed (see also Materials and Methods). Two candidate mt contigs having more than 100× read coverage were obtained (19,577 and 291,416 bp) (accession numbers: LC002801 and LC002802) by 49-kmer assembly. Physical link information from fosmid paired-end sequences (FPESs) confirmed contig structures from computational sequence assembly (fig. 1*A*). In addition, joining of the 3′-end of the approximately 19-kb contig and the 5′-end of the approximately 291-kb contig was supported by FPES. BLAST (Basic Local Alignment Search Tool) searches showed that the approximately 19-kb contig contains the *cox1* gene. The approximately 291-kb contig contains *cob*, *cox3*, and fragments of the LSU rRNA gene. Gene locations are explained in detail hereafter. Comparisons between the two contigs and the *S. minutum* genome assembly v1.0, using mapped FPES, showed that only scaffold 7473 (length: 15,538 bp) from genome assembly v1.0 (Shoguchi et al. 2013) was joined to the approximately 291-kb contig by more than 80 FPESs (fig. 1*A*). This suggested that nearly 40 kb of mtDNA had been identified. Estimation of the lengths of the two gaps was difficult. Accordingly, two bases (NN) were arbitrarily added between the two contigs and between the 291-kb contig and scaffold 7473 (see also fig. 1*A*). Comparison of the assembled mt genome with FPESs implies the presence of multiple recombinant mtDNA fragments, but our analysis suggests that *S. minutum* has a continuous mt genome of approximately 326 kb. Only simple repeats with fewer than 8 bp (∼1.49%) and low complexity (∼0.23%) were found in the mt genome assembly. The 49-bp repeats, which might be relevant to the assembly process, occurred fewer than four times in the approximately 326 kb.

### Transcriptomes of *Symbiodinium* mt Coding Genes

RNAseq reads were mapped onto the continuous genome (fig. 1*B*), revealing high coverage of *cox1*, *cob*, *cox3,* and the fragmented LSU gene (fig. 1*B*). Mapped data indicated the possibility of polycistronic expression. Mapping of reads with polyA or T in the 5′ sequence showed four major peaks for three protein-coding genes and the fragmented LSU. The highest peak is likely to be from the fragmented LSU gene, suggesting high expression and enhanced polyadenylation during RNA processing. Reads mapped from the transcription start site (TSS) library showed high coverage of multiple sites, suggesting multiple 5′ cleavage sites and transcripts with modified 5′-phosphate groups (fig. 1*B*). *Symbiodinium minutum* mt transcripts did not show evidence of RNA processing, such as 5′ oligo (U) caps of *O. marina* mt transcripts (Slamovits et al. 2007).

Edited RNA sites for transcripts of *cox1*, *cob*, and *cox3* were investigated using comparisons between assembled genomes and transcripts. A to G editing was found in 61% of the 72 sites, showing conservation between dinoflagellates (table 2; Lin et al. 2008). In addition, patterns of RNA editing-mediated amino acid substitutions correspond to previous report about another species (supplementary fig. S1, Supplementary Material online; Lin et al. 2008).

**Table 2:** RNA Editing Types in Three Mitochondrial Genes of *Symbiodinium minutum*

Gene	Transcriptome ID	No. of Edits (%)^a^	Editing Type							No. of Amino Acid Substitutions (%)
			A/G	G/A	C/U	U/C	G/C	U/G	A/C	
*cox1*	symbB1.comp234_c0_seq1	29/1,455 (2.0)	18	0	3	4	2	1	1	24/485 (4.9)
*cox3*	symbB1.comp4_c1_seq1, symbB1.EST_k37c20_2341	24/774 (3.1)	18	1	1	4	0	0	0	23/258 (8.9)^b^
*cob*	symbB1.EST_k37c20_4808	19/1,062 (1.8)	8	0	4	4	2	1	0	19/354 (5.4)^b^

^a^Edits in predicted coding sequences were counted.

^b^Including a signal from stop codon. For cox3, adenylated sequences may be used as stop signals.

Another unusual feature of dinoflagellate mt genes is the lack of canonical start and stop codons to direct the initiation and termination of translation (Norman and Gray 2001; Jackson et al. 2012; reviewed in Nash et al. 2008). We have characterized start and stop codons of *S. minutum* mt genes using manual alignments between genomic and transcriptomic sequences (supplementary fig. S2, Supplementary Material online). We found AUA (Ile) and AUU (Ile) at the 5′-end of *cox1* and AUU (Ile) in *cox3*. They are also candidates for start codons as mt genes in both ciliates and apicomplexans use AUA and AUU for this purpose (Feagin 1992; Edqvist et al. 2000). The *cob* gene in *S. minutum* contained both canonical start and stop codons; *cox3* contained a canonical stop codon resulting from polyA addition (supplementary fig. S2, Supplementary Material online), as reported in other dinoflagellates (Waller and Jackson 2009). *cox1* does not contain a stop codon (supplementary fig. S2, Supplementary Material online).

### Noncoding RNA Genes and Gene Map

In the apicomplexan *P. falciparum*, 39 RNA genes, including fragmented rRNA LSUs (15), SSUs (12), and uncharacterized small RNAs (12), have been identified (Feagin et al. 2012). These rRNA fragments are not arranged linearly, but synteny was conserved in *Plasmodium* (Vaidya and Mather 2009). It is suggested that this fragmentation occurred in the common ancestor of apicomplexans and dinoflagellates (Slamovits et al. 2007; Jackson et al. 2012). To predict fragmented rRNAs in the *S. minutum* mt genome, the most similar regions from *P. falciparum* (Feagin et al. 2012) were surveyed and aligned (table 3 and supplementary fig. S3, Supplementary Material online). Their alignments with interspersed regions of the *S. minutum* mt genome showed more than 50% similarity, corresponding to RNAseq reads from TSS libraries, and indicating the presence of multiple RNAs (fig. 1*B*). Comparisons of secondary structures for aligned sequences of RNA genes showed that the majority of predicted genes have stem-loop structures (supplementary fig. S3*B*, Supplementary Material online). Thus, the assembled *S. minutum* genome contains orthologs to genes in the *P. falciparum* mt genome (table 3 and supplementary fig. S3, Supplementary Material online).

**Table 3:** Predicted Genes in Mitochondrial Genome of *Symbiodinium minutum*

Gene	Subunit Order	Predicted Location	Orientation to Scaffold	Similarity to *Plasmodium falciparum* Gene (%)^a^
*cox1*		5809–7248	+	916/1,441 (63)
*cox3*		186587–187332	+	405/771 (52)
*cob*		197602–198718	+	688/1131 (60)
*SSUA*	S4	177279–177354	+	57/80 (71)
*SSUB*	S6	236311–236394	+	48/86 (55)
*SSUD*	S10	176902–176959	−	37/63 (58)
*SSUE*	S11	221699–221724	+	19/26 (73)
*SSUF*	S12	170409–170456	+	31/48 (64)
*LSUA*	L1	105339–105493	−	89/158 (56)
*LSUB*	L3	38813–38831	−	17/19 (89)
*LSUC*	L4	73563–73580	−	17/18 (94)
*LSUD*	L8	222761–222836	+	55/76 (72)
*LSUE*	L9	193381–193573	+	149/195 (76)
*LSUF*	L11	279828–279907	+	55/80 (68)
*LSUG*	L12	278801–278900	−	74/100 (74)
*RNA1*	L6	317063–317147	+	54/88 (61)
*RNA2*	L2	60688–60729	+	26/42 (61)
*RNA3*	L7	34514–34593	−	45/81 (55)
*RNA4*		220439–220506	−	39/68 (57)
*RNA5*	S9	138204–138280	+	48/80 (60)
*RNA6*	L15	14596–14626	−	27/33 (81)
*RNA7*		56199–56266	+	53/69 (76)
*RNA8*	S5	106227–106279	−	30/53 (56)
*RNA9*	S8	281819–281866	+	34/50 (68)
*RNA10*	L13	217165–217255	+	59/92 (64)
*RNA11*	L5	73434–73479	−	29/46 (63)
*RNA12*	S2	191852–191892	+	30/41 (73)
*RNA13*	L10	256591–256614	−	17/24 (70)
*RNA14*	S1	31151–31177	+	21/27 (77)
*RNA15*		253421–253447	−	19/27 (70)
*RNA16*		51961–51991	−	21/31 (67)
*RNA17*	S3	76949–76985	+	24/37 (64)
*RNA18*	L14	300291–300312	+	17/22 (77)
*RNA19*	S7	308063–308089	−	21/27 (77)
*RNA20*		242197–242225	+	20/29 (68)
*RNA21*		122845–122864	−	16/20 (80)
*RNA22*		57665–57699	+	24/35 (68)
*RNA23t*		186459–186487	−	20/29 (68)
*RNA24t*		23626–23665	−	27/40 (67)
*RNA25t*		302697–302717	+	16/21 (76)
*RNA26t*		186374–186415	−	29/43 (67)
*RNA27t*		278662–278712	−	36/52 (69)

Note.—Gene names and subunit order refer to Feagin et al. (2012). L and S indicate LSU and SSU, respectively.

^a^Genes are from *P. falciparum* M76611 (Feagin et al. 2012). Alignments are shown in supplementary figure S3, Supplementary Material online.

tRNA genes were not found in the *S. minutum* mt genome using tRNA scan. So far no studies of dinoflagellate or apicomplexan mtDNAs have identified any tRNA genes, suggesting that tRNAs have been imported from the nuclear genome, as was reported for the apicomplexan, *Toxoplasma gondii* (Esseiva et al. 2004).

Interestingly, two LSU fragments, L4 and L5, map onto neighboring regions of the *S. minutum* mt genome with fewer than 100 bp between them (table 3 and supplementary fig. S4, Supplementary Material online). The L4–L5 arrangement in *S. minutum*, corresponding to the continuous large rRNA sequence order, appears to be evolutionarily conserved. Secondary structure prediction for L4 and L5 sequences yields a very stable, double-stranded form; however, the predicted structure varies depending on which secondary structure prediction program is used (supplementary fig. S3*C* and *D*, Supplementary Material online). Genes with unknown functions, RNA 23 t and RNA 26 t, are close, separated by only an approximately 40-bp intergenic sequence. Conserved, fragmented LSUs and SSUs may be cleaved accurately by small RNAs, such as RNA 23 t and RNA 26 t.

**Fig. 2.—:**
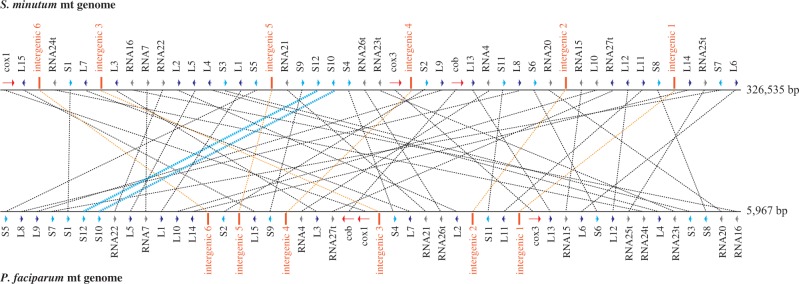
Mitochondrial gene order comparisons between *S. minutum* and *P. falciparum*. Genes from the *S. minutum* mt genome (∼326 kb) to the upper are joined to those of *P. falciparum*. The gene order of S10 and S12 was the same in mt genomes of both *P. falciparum* and *S. minutum* (aqua lines)*,* showing minimal conservation of gene order. Sequence similarities from intergenic regions of the *P. falciparum* mt genome are indicated by orange lines. Details for the *S. minutum* mt genome map are shown in supplementary figure S4, Supplementary Material online.

Comparisons of mt gene arrangements between *Symbiodinium* and *Plasmodium* showed only one microsyntenic region, which has the same gene arrangement on S12 and S10 (fig. 2 and supplementary fig. S4, Supplementary Material online), suggesting that the fragmentation occurred in the common ancestor of apicomplexans and dinoflagellates and that genome rearrangements in these lineages were very frequent. Thus, this basic information is valuable for possible functional analysis of dinoflagellate mt genomes.

### Unknown Noncoding Regions and Possible Expansion in the Dinoflagellate Lineage

Our analysis confirms that noncoding sequences of the *Symbiodinium* mt genome have been expanded, raising the question as to where the expanded sequences originated. Enormous expansion of intergenic content in the mt genomes of seed plants (200–2,900 kb) has been reported (Mower et al. 2012), and repeated proliferation of “selfish” DNA has contributed overwhelmingly to these expansions (Chaw et al. 2008). Highly repetitive sequences were not found in the mt genome of *S. minutum*. In addition, when compared with other dinoflagellate mt sequences, the *S. minutum* mt sequences suggest additional RNA fragments or pseudogenes (supplementary fig. S5, Supplementary Material online), but these were not identified.

To find conserved secondary structures of potential RNA genes, each chopped 300-bp sequence from the mt genome was employed as a query sequence to perform RNA homology searches using Infernal (Nawrocki and Eddy 2013). Twenty-two sequences showed similarity to reported sequences in the Rfam database, including microRNAs and LSUs (Nawrocki et al. 2015) and suggest the presence of unknown RNA genes (supplementary table S1, Supplementary Material online). Unexpectedly, possible secondary structures for these genes included a stem of more than 20 bp (supplementary fig. S6, Supplementary Material online). Although we did not find sequence similarities to reported small, IRs of dinoflagellate mtDNA (reviewed in Waller and Jackson 2009) in the *S. minutum* mtDNA, the result suggests that structures comprising IRs are conserved characters among dinoflagellates mtDNAs.

Transcriptional control of the alveolate mt genome is not clear (Gray et al. 2004; Waller and Jackson 2009). Our RNAseq reads support the possibility of polycistronic expression. To detect conserved intergenic regions, similarities to the six intergenic sequences of *P. falciparum* and mtDNA sequences of dinoflagellates were surveyed. Interestingly, our analysis showed that intergenic sequences of *P. falciparum* have similarities to the mt genome of *S. minutum* at the same level as comparisons between rRNA sequences. (fig. 2 and supplementary fig. S7, Supplementary Material online).

It is very interesting to examine how organelle genomes of dinoflagellates evolved different structures, given the large variety of structures between plastid and mt genomes of *Symbiodinium*. Although the plastid genome has undergone reconfiguration to a compact DNA minicircle (∼1.8–3.0 kb) with its own regulatory regions (Mungpakdee et al. 2014), the mt genome appears greatly expanded and fragmented.

## Materials and Methods

### Genome Assembly

Genomic DNA sequences from cloned and cultured *S. minutum* Mf1.05 b.01 (clade type: B1) were obtained previously (Shoguchi et al. 2013). To obtain longer mt contigs, using Velvet (version 1.2.08) software (Zerbino and Birney 2008) for illumina paired-end reads (6.8 Gb from a polymerase chain reaction-free library, accession number: DRX003100), calculations of 20 patterns were performed on a combination of ten kmer parameters (kmer size: 27, 31, 35, 37, 41, 43, 45, 47, 49, and 51) using two coverage threshold values (>50 and >100). The 151,553 FPESs with approximately 2.1× coverage of the whole genome (Hattori et al. 2000; Shoguchi et al. 2013) (deposited in DNA Data Bank of Japan, accession numbers: GA453877–GA605429) were mapped onto mt contigs using BLASTN and relationships between contigs were examined.

The assembled mt genome was also compared with FPESs and assembly version 1 scaffolds from contigs based on Roche 454 reads (GenBank ID: BASF00000000.1; see also http://marinegenomics.oist.jp/genomes/gallery, last accessed September 1, 2014) (Koyanagi et al. 2013; Shoguchi et al. 2013).

### Transcriptome Mapping

Transcriptome reads deposited at DRP000944 were mapped onto the assembled mt genome using Bowtie 2 (version 2.1.0) software (Langmead and Salzberg 2012). Detection of RNA editing for *cob*, *cox1,* and *cox3* was basically performed in the same manner as for plastid transcripts (Mungpakdee et al. 2014). Differences between DNA and RNA were detected by aligning transcriptome contigs to a scaffold. RNAseq reads were mapped onto mt transcriptome contigs using TopHat (Trapnell et al. 2009) and accuracy was confirmed. Reads from a TSS library (Yamashita et al. 2011; Shoguchi et al. 2013) were mapped using Bowtie 2, as described in Mungpakdee et al. (2014).

### Data Analysis Software and Sequences Used for Comparisons

Repeats in the mt genome assembly were detected using RepeatMasker in default mode (http://www.repeatmasker.org). tRNAscan-SE (with default parameters in organellar mode) (Schattner et al. 2005) was used to find tRNA genes in the mt genome and transcriptome contigs. Sequence alignments between *Symbiodinium* and *Plasmodium* were performed using GENETYX-MAC version 17 and BLASTN. RNAfold (http://rna.tbi.univie.ac.at/cgi-bin/RNAfold.cgi; Zuker and Stiegler 1981) and CentroidFold (http://www.ncrna.org/centroidfold/; Sato et al. 2009) with initial settings were used for prediction of RNA secondary structure. We prepared chopped 300-bp sequences from the mt genome with 100-bp overlap sequences. RNA homology searches for each of the 300-bp sequences were performed using infernal (INFERence of RNA ALignment) using default parameters (Nawrocki and Eddy 2013). The probability cutoff value (*E* value) was set at 0.001.

mt genome sequences used for comparisons have the following accession numbers: “HE610722–HE610773” for *Hematodinium* sp., “AB265207–AB265210 and AB374233–AB374251” for *Alexandrium catenella*, “JX001584–JX001600” for *Durinskia baltica*, “EF442995–EF443047 and AM773790–AM773803” for *Karlodinium micrum*, “JX001601–JX001608” for *Kryptoperidinium foliaceum*, “EF680822–EF680839” for *O. marina*, “M76611” for *P. falciparum,* “KF651061” for Alveolata sp. 1 JJ-2013 (colponemid-like Peru), which is a new species, *Acavomonas peruviana* (Tikhonenkov et al. 2014), and “AF396436” for *T. thermophile*.

## Supplementary Material

Supplementary figures S1–S7 and table S1 are available at *Genome Biology and Evolution* online (http://www.gbe.oxfordjournals.org/).

Supplementary Data

## References

[evv137-B1] Burger G 2000 Complete sequence of the mitochondrial genome of *Tetrahymena pyriformis* and comparison with *Paramecium aurelia* mitochondrial DNA. J Mol Biol. 297:365-380. 10715207 10.1006/jmbi.2000.3529

[evv137-B2] Chaput H Wang Y Morse D 2002 Polyadenylated transcripts containing random gene fragments are expressed in dinoflagellate mitochondria. Protist 153:111-122. 12125753 10.1078/1434-4610-00090

[evv137-B3] Chaw SM 2008 The mitochondrial genome of the gymnosperm *Cycas taitungensis* contains a novel family of short interspersed elements, Bpu sequences, and abundant RNA editing sites. Mol Biol Evol. 25:603-615. 18192697 10.1093/molbev/msn009

[evv137-B4] Coffroth MA Santos SR 2005 Genetic diversity of symbiotic dinoflagellates in the genus *Symbiodinium*. Protist 156:19-34. 16048130 10.1016/j.protis.2005.02.004

[evv137-B6] Edqvist J Burger G Gray MW 2000 Expression of mitochondrial protein-coding genes in *Tetrahymena pyriformis*. J Mol Biol. 297:381-393. 10715208 10.1006/jmbi.2000.3530

[evv137-B8] Esseiva AC Naguleswaran A Hemphill A Schneider A 2004 Mitochondrial tRNA import in *Toxoplasma gondii*. J Biol Chem. 279:42363-42368. 15280394 10.1074/jbc.M404519200

[evv137-B9] Feagin JE 1992 The 6-kb element of *Plasmodium falciparum* encodes mitochondrial cytochrome genes. Mol Biochem Parasitol. 52:145-148. 1320735 10.1016/0166-6851(92)90046-m

[evv137-B10] Feagin JE 2012 The fragmented mitochondrial ribosomal RNAs of *Plasmodium falciparum*. PLoS One 7:e38320. 22761677 10.1371/journal.pone.0038320 PMC3382252

[evv137-B11] Gray MW Lang BF Burger G 2004 Mitochondria of protists. Annu Rev Genet. 38:477-524. 15568984 10.1146/annurev.genet.37.110801.142526

[evv137-B12] Hattori M 2000 The DNA sequence of human chromosome 21. Nature 405:311-319. 10830953 10.1038/35012518

[evv137-B13] Hikosaka K 2012 Novel type of linear mitochondrial genomes with dual flip-flop inversion system in apicomplexan parasites, *Babesia microti* and *Babesia rodhaini*. BMC Genomics 13:622. 23151128 10.1186/1471-2164-13-622 PMC3546061

[evv137-B14] Imanian B Keeling PJ 2007 The dinoflagellates *Durinskia baltica* and *Kryptoperidinium foliaceum* retain functionally overlapping mitochondria from two evolutionarily distinct lineages. BMC Evol Biol. 7:172. 17892581 10.1186/1471-2148-7-172 PMC2096628

[evv137-B15] Imanian B Pombert JF Dorrell RG Burki F Keeling PJ 2012 Tertiary endosymbiosis in two dinotoms has generated little change in the mitochondrial genomes of their dinoflagellate hosts and diatom endosymbionts. PLoS One 7:e43763. 22916303 10.1371/journal.pone.0043763 PMC3423374

[evv137-B16] Jackson CJ 2007 Broad genomic and transcriptional analysis reveals a highly derived genome in dinoflagellate mitochondria. BMC Biol. 5:41. 17897476 10.1186/1741-7007-5-41 PMC2151934

[evv137-B17] Jackson CJ Gornik SG Waller RF 2012 The mitochondrial genome and transcriptome of the basal dinoflagellate *Hematodinium* sp.: character evolution within the highly derived mitochondrial genomes of dinoflagellates. Genome Biol Evol. 4:59-72. 22113794 10.1093/gbe/evr122 PMC3268668

[evv137-B18] Jackson CJ Waller RF 2013 A widespread and unusual RNA trans-splicing type in dinoflagellate mitochondria. PLoS One 8:e56777. 23437234 10.1371/journal.pone.0056777 PMC3577742

[evv137-B19] Janouškovec J 2013 Colponemids represent multiple ancient alveolate lineages. Curr Biol. 23:2546-2552. 24316202 10.1016/j.cub.2013.10.062

[evv137-B21] Kamikawa R Inagaki Y Sako Y 2007 Fragmentation of mitochondrial large subunit rRNA in the dinoflagellate *Alexandrium catenella* and the evolution of rRNA structure in alveolate mitochondria. Protist 158:239-245. 17291829 10.1016/j.protis.2006.12.002

[evv137-B22] Kamikawa R Nishimura H Sako Y 2009 Analysis of the mitochondrial genome, transcripts, and electron transport activity in the dinoflagellate *Alexandrium catenella* (Gonyaulacales, Dinophyceae). Phycol Res. 57:1-11.

[evv137-B23] Koyanagi R 2013 MarinegenomicsDB: an integrated genome viewer for community-based annotation of genomes. Zool Sci. 30:797-800. 24125644 10.2108/zsj.30.797

[evv137-B24] Lang BF Gray MW Burger G 1999 Mitochondrial genome evolution and the origin of eukaryotes. Annu Rev Genet. 33:351-397. 10690412 10.1146/annurev.genet.33.1.351

[evv137-B25] Langmead B Salzberg SL 2012 Fast gapped-read alignment with Bowtie 2. Nat Methods. 9:357-359. 22388286 10.1038/nmeth.1923 PMC3322381

[evv137-B27] Lin S Zhang H Gray MW 2008 RNA editing in dinoflagellates and its implications for the evolutionary history of the editing machinery. In: Smith HC, editor RNA and DNA editing: molecular mechanisms and their integration into biological systems. Hoboken (NJ): John Wiley & sons, Inc p. 280–309.

[evv137-B28] Lin S Zhang H Spencer DF Norman JE Gray MW 2002 Widespread and extensive editing of mitochondrial mRNAS in dinoflagellates. J Mol Biol. 320:727-739. 12095251 10.1016/s0022-2836(02)00468-0

[evv137-B30] Mower JP Case AL Floro ER Willis JH 2012 Evidence against equimolarity of large repeat arrangements and a predominant master circle structure of the mitochondrial genome from a monkeyflower (*Mimulus guttatus*) lineage with cryptic CMS. Genome Biol Evol. 4:670-686. 22534162 10.1093/gbe/evs042 PMC3381676

[evv137-B31] Mungpakdee S 2014 Massive gene transfer and extensive RNA editing of a symbiotic dinoflagellate plastid genome. Genome Biol Evol. 6:1408-1422. 24881086 10.1093/gbe/evu109 PMC4079212

[evv137-B32] Nash EA 2007 Organization of the mitochondrial genome in the dinoflagellate *Amphidinium carterae*. Mol Biol Evol. 24:1528-1536. 17440175 10.1093/molbev/msm074

[evv137-B33] Nash EA Nisbet RE Barbrook AC Howe CJ 2008 Dinoflagellates: a mitochondrial genome all at sea. Trends Genet. 24:328-335. 18514360 10.1016/j.tig.2008.04.001

[evv137-B34] Nawrocki EP Eddy SR 2013 Infernal 1.1: 100-fold faster RNA homology searches. Bioinformatics 29:2933-2935. 24008419 10.1093/bioinformatics/btt509 PMC3810854

[evv137-B35] Nawrocki EP 2015 Rfam 12.0: updates to the RNA families database. Nucleic Acids Res. 43:D130-D137. 25392425 10.1093/nar/gku1063 PMC4383904

[evv137-B36] Norman JE Gray MW 1997 The cytochrome oxidase subunit 1 gene (cox1) from the dinoflagellate, *Crypthecodinium cohnii*. FEBS Lett. 413:333-338. 9280308 10.1016/s0014-5793(97)00938-1

[evv137-B37] Norman JE Gray MW 2001 A complex organization of the gene encoding cytochrome oxidase subunit 1 in the mitochondrial genome of the dinoflagellate, *Crypthecodinium cohnii*: homologous recombination generates two different cox1 open reading frames. J Mol Evol. 53:351-363. 11675595 10.1007/s002390010225

[evv137-B56] Patron NJ Waller RF Archibald JM Keeling PJ 2005 Complex protein targeting to dinoflagellate plastids. J Mol Biol. 348:1015-1024. 15843030 10.1016/j.jmb.2005.03.030

[evv137-B38] Pochon X Putnam HM Gates RD 2014 Multi-gene analysis of *Symbiodinium* dinoflagellates: a perspective on rarity, symbiosis, and evolution. PeerJ. 2:e394. 24883254 10.7717/peerj.394 PMC4034598

[evv137-B39] Rycovska A Valach M Tomaska L Bolotin-Fukuhara M Nosek J 2004 Linear versus circular mitochondrial genomes: intraspecies variability of mitochondrial genome architecture in *Candida parapsilosis*. Microbiology 150:1571-1580. 15133118 10.1099/mic.0.26988-0

[evv137-B40] Sato K Hamada M Asai K Mituyama T 2009 CENTROIDFOLD: a web server for RNA secondary structure prediction. Nucleic Acids Res. 37:W277–W280. 19435882 10.1093/nar/gkp367 PMC2703931

[evv137-B41] Schattner P Brooks AN Lowe TM 2005 The tRNAscan-SE, snoscan and snoGPS web servers for the detection of tRNAs and snoRNAs. Nucleic Acids Res. 33:W686–W689. 15980563 10.1093/nar/gki366 PMC1160127

[evv137-B42] Shinzato C Mungpakdee S Satoh N Shoguchi E 2014 A genomic approach to coral-dinoflagellate symbiosis: studies of *Acropora digitifera* and *Symbiodinium minutum*. Front Microbiol. 5:336. 25071748 10.3389/fmicb.2014.00336 PMC4083563

[evv137-B43] Shoguchi E 2013 Draft assembly of the *Symbiodinium minutum* nuclear genome reveals dinoflagellate gene structure. Curr Biol. 23:1399-1408. 23850284 10.1016/j.cub.2013.05.062

[evv137-B44] Slamovits CH Saldarriaga JF Larocque A Keeling PJ 2007 The highly reduced and fragmented mitochondrial genome of the early-branching dinoflagellate *Oxyrrhis marina* shares characteristics with both apicomplexan and dinoflagellate mitochondrial genomes. J Mol Biol. 372:356-368. 17655860 10.1016/j.jmb.2007.06.085

[evv137-B45] Sloan DB 2012 Rapid evolution of enormous, multichromosomal genomes in flowering plant mitochondria with exceptionally high mutation rates. PLoS Biol. 10:e1001241. 22272183 10.1371/journal.pbio.1001241 PMC3260318

[evv137-B57] Trapnell C Pachter L Salzberg SL 2009 TopHat: discovering splice junctions with RNA-Seq. Bioinformatics. 25:1105-1111. 19289445 10.1093/bioinformatics/btp120 PMC2672628

[evv137-B47] Tikhonenkov DV 2014 Description of *Colponema vietnamica* sp.n. and *Acavomonas peruviana* n. gen. n. sp., two new alveolate phyla (Colponemidia nom. nov. and Acavomonidia nom. nov.) and their contributions to reconstructing the ancestral state of alveolates and eukaryotes. PLoS One 9:e95467. 24740116 10.1371/journal.pone.0095467 PMC3989336

[evv137-B48] Vaidya AB Mather MW 2009 Mitochondrial evolution and functions in malaria parasites. Annu Rev Microbiol. 63:249-267. 19575561 10.1146/annurev.micro.091208.073424

[evv137-B49] Waller RF Jackson CJ 2009 Dinoflagellate mitochondrial genomes: stretching the rules of molecular biology. Bioessays 31:237-245. 19204978 10.1002/bies.200800164

[evv137-B50] Yamashita R 2011 Genome-wide characterization of transcriptional start sites in humans by integrative transcriptome analysis. Genome Res. 21:775-789. 21372179 10.1101/gr.110254.110 PMC3083095

[evv137-B51] Zerbino DR Birney E 2008 Velvet: algorithms for de novo short read assembly using de Bruijn graphs. Genome Res. 18:821-829. 18349386 10.1101/gr.074492.107 PMC2336801

[evv137-B52] Zhang H Bhattacharya D Maranda L Lin S 2008 Mitochondrial cob and cox1 genes and editing of the corresponding mRNAs in *Dinophysis acuminata* from Narragansett Bay, with special reference to the phylogenetic position of the genus *Dinophysis*. Appl Environ Microbiol. 74:1546-1554. 18165361 10.1128/AEM.02103-07 PMC2258633

[evv137-B53] Zhang H Lin S 2002 Detection and quantification of *Pfiesteria piscicida* by using the mitochondrial cytochrome b gene. Appl Environ Microbiol. 68:989-994. 11823251 10.1128/AEM.68.2.989-994.2002 PMC126730

[evv137-B54] Zhang H Lin S 2005 Mitochondrial cytochrome b mRNA editing in dinoflagellates: possible ecological and evolutionary associations? J Eukaryot Microbiol. 52:538-545. 16313447 10.1111/j.1550-7408.2005.00060.x

[evv137-B55] Zuker M Stiegler P 1981 Optimal computer folding of large RNA sequences using thermodynamics and auxiliary information. Nucleic Acids Res. 9:133-148 6163133 10.1093/nar/9.1.133 PMC326673

