# Supplementary material for: The Large Mitochondrial Genome of Symbiodinium minutum Reveals Conserved Noncoding Sequences between Dinoflagellates and Apicomplexans
Source: Genome Biol Evol. 2015 Jul 20;7(8):2237–44. doi: 10.1093/gbe/evv137 (PMC4558855; doi:10.1093/gbe/evv137)
Supplement: Supplementary Data [file supp_7_8_2237__index.html]

The Large Mitochondrial Genome of Symbiodinium minutum Reveals Conserved Noncoding Sequences between Dinoflagellates and Apicomplexans — Supplementary Data 

# The Large Mitochondrial Genome of *Symbiodinium minutum* Reveals Conserved Noncoding Sequences between Dinoflagellates and Apicomplexans

## Supplementary Data

files

- Supplementary Data - zip file
