# Supplementary material for: The Large Mitochondrial Genome of Symbiodinium minutum Reveals Conserved Noncoding Sequences between Dinoflagellates and Apicomplexans
Source: Genome Biol Evol. 2015 Jul 20;7(8):2237–44. doi: 10.1093/gbe/evv137 (PMC4558855; doi:10.1093/gbe/evv137)
Supplement: Supplementary Data [file supp_evv137_suppl_data.zip › Supple_figs1,2,3,4,5-Shoguchi_et_al.pdf]

supplementary fig. 1

```
           1        10        20        30        40        50        60        70        80        90       100
      cox1 INLLTINFLFHSLVKNCNHKGLGIYYLLSGFIFGISGTLISVLIRIELYSSGNRIISPENONFYNISITLHGFLMIFFLVMPGLFGGFGNYFVPIFOGSP  100
cox1 edits                     R                      M                     V      L

      cox1 EVVYPRVNNFSILILFLSYLFLILSLISEFGGGTGWTLYPPLSTSFMTLSPSSTGNLIFGLIISGISSSLTSLNFWTTIHFLRSYYLILSSIPLFPWAFL  200
cox1 edits                                                      V                      V        S

      cox1 ITAFMLLLTLPILSGTLLLILGDLHSNTLFFDPIFGGDPIFYQHLFWFFGHPEVYILIIPAFGIISIIISGILQLIIFANQSMIFAMSSISLLGGLVWGH  300
cox1 edits            V       V             V     VL                      V   V   VS

      cox1 HMYTVGLESDTRAYFTGVTILISLPTGTKIFNWLFTYLSNPPLLHLRITSVFLSHLFLLMFTVGGSTGIILGNGAVDLGLHDTYYVVAHFHFVLSLGAII  400
cox1 edits                                                                     V         A                   V

      cox1 AIFSGIILNGEKIVATKNLLLSSSCTLSLYHLHLIFIGILLTFSPMHFLGFNLMPRRIPSFPDSFHSWNSLSSIGSGITFLSFST
cox1 edits       V          S                V V                                          L    M
```

COX1 (PF00115)

```
           1        10        20        30        40        50        60        70        80        90       100
      cox3 INTELLFYYLYLLVFWKSLHLEVTSDLFCFLINTHHLLIFALIIILFIFTGFNIYCWTGIYFS*NYFLILFHLNFGLIYSFHIWSRDLLREFTKKYEVLL  100
cox3 edits                        A GS  L   A Y      VV             A     WS S                              I

      cox3 IIFFLLFGGFLVSEALLFVSFFWTSFHLLSSPTAGMWLWEAFYLEDPCELTFANTLLLSNAAISLGNAFINLEISSEYIIFFTLWSFFLSSLFISLQIKE  200
cox3 edits MVL                    A                                      V   G   S     G     A

      cox3 FRILALSINDSLYSSLFFFLTGLHFFHLSIGLILLIYFYYVGFFVYFMLSWTSFNKI*
cox3 edits   V
```

COX3 (PF00510)

```
          1        10        20        30        40        50        60        70        80        90       100
      cob MKSHLQSYPCPLIINYFWNLGFLLGITILLQIISGIFLGLHYTSDINSAYFSIFFIIREIYYGWCLRYLHSNGSSFVFLFIFLHLGRAISYGSYFYNPNT  100
cob edits                          VA                               V        F

      cob WFSGIIIIFFLMGTAFMGYVLPLGQMSLWGVTVITNLLSAFPSLIEWLCGGHYIYNPTFKRFFVFHFLFPFLLCGFLLYHIFNLHFLSSNNPLRNSTNNK  200
cob edits        V     I                              V      YC H             L             S

      cob IAFLPFIISKDFYGMILISYLYLLQIHFGFSSFSHPDNALEACGLLTPLHIVPEWYFLCQYAMLKAVPNKNAGFIVLLTSIFVLFYFMRSLSISFYFIVW  300
cob edits            L          F         L          A R                                               V

      cob VSSRFNSFFVILWFLVFISLIWIGGQFPVDNFLSYGRILTLYYYYFLICILLS*
cob edits                                    A
```

Cytochrom_B_N_2 (PF13631)

Cytochrom_B_C (PF00032)

```
Cox1          5′                                                                           3′
Kmic            FNKSSQRIFFLSLVKNCNHK-nnnnnn-FPDTFH-WNFLSSIGSGVTLLSFGMLKKK------------
Ppis            ---------FISLLKNCNHK-nnnnnn-FPDSFHSWNFLSSIGSGITLLSFGFLKKK------------
Acat            LNS-----FSLSHVKNCNHK-nnnnnn-FPDSFHSWNFLSSIGSGITLLS-TMLKKK------------
Smi-RNA         INLLTINFLFHSLVKNCNHK-nnnnnn-FPDSFHSWNSLSSIGSGITLLSFSMKK--------------
Smi-DNA         INLLTINFLFHSLVKNCNHK-nnnnnn-FPDSFHSWNSLSSIGSGITFLSFSTNGGAYKWKNRGPSTS.
Hem             FHTLQ----IYSLTKNCNHK-nnnnnn-FPNYFNSWNYLSSIGSGITLICWFSLKKK------------
                           *  ******        **: *: ** *******:*::.

Cob           5′                                                          3′
Acat            -----LYFVLLMKSHFQSY-nnnnnn-FISYIRLLTINYYFLIISILILKKK
Kmic            -----LHFVLLMKSHLQSY-nnnnnn-FLSYARILTLDFYFLLICISFSKKK
Smi-RNA         -----LYFLLLMKSHLQSY-nnnnnn-FLSYARILTLYYYYFLICILLS.--
Smi-DNA         -----LYFLLLMKSHLQSY-nnnnnn-FLSYGRILTLYYYYFLICILLS.--
Ppis            CIYRITYFVLLMKSHLQSY-nnnnnn-FLSYARILTLYYYFLLMCILFSKK-
Hem              PFK-FQYLFLIKSHLQSY-nnnnnn-FISYGRILTLFYYFIIIYLLFEKKK
                       :::*:***:***        *:** *:**: :*:::: : :

Cox3          5′                                                               3′
Smi-RNA         FINTELLFYYLYLLVFWK-SL-nnnnnn-ILLIYFYYVGFFVYFM-LSWTSFNKI.
Acat            F----IVFISAWRLVFWN-SI-nnnnnn-LQLFYWHFLEILWIFIFLVFYKSKKK-
Symsp           F----FRWY-SFTLVFWK-SK-nnnnnn-LQLFYWHFLEILWLFIFLVFYL-KKK-
Hem             ---------------FYNLNI-nnnnnn-LQLIYWHFIELLWLFIYMVLYSYQKKK
Kmic            Y----NFGNQLLYFGFSN-SI-nnnnnn-LQNFYWHFLEILWLFIFLFLYSLKKK-
                               * : .         :  :*:::: ::  *: :     :*
```

supplementary fig. 3

```
1st Nucleotide Sequence
  File Name       : S_minutum_mtDNA-v1.seq
  Sequence Size   : 326535

2nd Nucleotide Sequence
  File Name       : cox1_lcl|M76611.1[2037-3479]
  Sequence Size   : 1443

Query Range: 5809 - 7248
Sbjct Range: 5 - 1425
Identity: 916 / 1441 (63%)
Similarity: 916 / 1441 (63%)
Gaps: 21 / 1441 (1%)
Strand: Plus / Plus

Query  5809  ATTAATTTCCTATTTCATTCACTAGTTAAAAATTGTAATCATAAAGGCTTAGGAATCTAT  5868
             ***  ***        *******  *** ********* ******   ****** * **
Sbjct     5  ATTGTTTTAAATAGATATTCACTTATTACAAATTGTAACCATAAAACTTTAGGATTATAC  64

Query  5869  TATTTATTATCTGGATTCATCTTTGGAATCTCCGGTACATTAATATCAGTCCTTATAAGA  5928
             *******  * *  ***  * ***** *  *  **    *** *******  *  ** *
Sbjct    65  TATTTATGGTTTTCATTTTTATTTGGTAGTTATGGATTTTTATTATCAGTAATACTACGT  124

Query  5929  ATAGAATTATATTCTTCAGGAAATAGGATTATATCTCCAGAAAACCAGAACTTCTATAAT  5988
             *  ***************      ** ** **  * * ******    **  * ******
Sbjct   125  ACTGAATTATATTCTTCATCTTTAAGAATAATTGCACAAGAAAATGTAAATCTATATAAT  184

Query  5989  ATAAGCATTACATTGCATGGCTTTCTTATGATTTTCTTTTTAGTAATGCCTGGCTTGTTT  6048
             ** *   ***** * ** **  *  ********** **     ******* ** ** **
Sbjct   185  ATGATATTTACAATTCACGGAATAATTATGATTTTTTTCAATATAATGCCAGGATTATTC  244

Query  6049  GGAGGATTTGGAAATTATTTTGTACCTATCTTTCAAGGGTCTCCAGAAGTGGTATATCCT  6108
             *********** ***** *** ******* **    ** ********* * * *******
Sbjct   245  GGAGGATTTGGTAATTACTTTCTACCTATTTTATGTGGATCTCCAGAATTAGCATATCCT  304

Query  6109  AGAGTCAATAATTTTTCTATCTTAATTCTTTTCCTTTCATATCTTTTCCTAATCCTTTCT  6168
             *** * **** * * *** *  *  * *      ** * * * ****  * **  * ***
Sbjct   305  AGAATTAATAGTATATCTTTACTGTTACAACCAATTGCTTTTGTTTTAGTTATATTATCT  364

Query  6169  TTAATCTCAGAATTTGGAGGTGGTACAGGGTGGACGCTCTACCCACCATTATCCACTTCT  6228
                    ********** ***** ** ** *****  * ** *********   ** ***
Sbjct   365  ACTGCAGCAGAATTTGGTGGTGGAACTGGATGGACTTTATATCCACCATTAAGTACATCT  424

Query  6229  TTTATGACTTTATCACCTTCAAGTACAGGAAATCTTATATTTGGATTAATAATCTCTGGT  6288
             ** *** * ***** ***  *  *  **      * ** ***** *** ** * *****
Sbjct   425  TTAATGTCATTATCTCCTGTAGCTGTAGATGTAATAATTTTTGGTTTATTAGTATCTGGA  484

Query  6289  ATATCTTCATCTCTTACATCTCTTAACTTTTGGACAACAATTCATTTTCTGAGATCTTAT  6348
              *  **     * *  * **  * ** ***   ** *** *      * * *** *  *
Sbjct   485  GTCGCTAGTATTATGTCTTCATTAAATTTTATTACTACAGTAATGCATTTAAGAGCAAAA  544

Query  6349  TATCTGATATTATCTTCTATCCCAT-TATTTCCTTGGGCTTTCTTGATTACAGCTTTCAT  6407
                 * * * * *  * ***    * * * * * *** * **  * ****** *    **
Sbjct   545  GGATTAACACT-TGGTATATTAAGTGTTTCTACATGGTCATTGATCATTACATCAGGAAT  603

Query  6408  GCTTTTATTAACATTACCAATCTTATCTGGTACACTTCTTTTAATATTGGGTGATCTTCA  6467
             * * **  ***** ****  * *** ****   * *  *  *  ****    **  * **
Sbjct   604  GTTATTGCTAACACTACCGGTTTTAACTGGAGGAGTATTAATGTTATTATCAGACTTACA  663

Query  6468  TTCAAATACACTTTTCTTTGATCCAATATTTGGAGGAGATCCTATATTCTATCAACATTT  6527
             **  *****  * ** ***** **** ***** ********* ***** ***********
Sbjct   664  TTTTAATACTTTATTTTTTGACCCAACATTTGCAGGAGATCCAATATTATATCAACATTT  723

Query  6528  ATTTTGGTTTTTTGGACATCCAGAAGTTTACATATTAATAATTCCTGCATTTGGGATCAT  6587
             *** ***************** ***** ***** ****** * ***** *****  * **
Sbjct   724  ATTCTGGTTTTTTGGACATCCTGAAGTATACATTTTAATATTACCTGCTTTTGGAGTAAT  783
```

```
Query  6588  TTCCATAATAATTTCTGGGATTTTACAGTTAATAATCTTTGCTAACCAATCAATGATCTT  6647
             *       ********   * **       **   * **** *** ***** *****  *
Sbjct   784  TAGTCATGTAATTTCTACTAATTATTGCAGAAATCTATTTGGTAATCAATCTATGATACT  843

Query  6648  TGCCATGTCATCTATTTCTCTTCTTGGAGGTCTTGTTTGGGGACATCATATGTATACTGT  6707
             *** ***  ** ***  ** ** * *** *  * ** **** ************ ***
Sbjct   844  TGCTATGGGATGTATAGCTGTTTTAGGAAGCTTAGTATGGGTACATCATATGTACACTAC  903

Query  6708  AGGTTTAGAAAGTGATACAAGAGCTTATTTTACAGGAGTTACAATCTTAATATCCTTACC  6767
              *********  ****** **************      *** ** ********  ****
Sbjct   904  TGGTTTAGAAGTTGATACTAGAGCTTATTTTACTTCGACTACCATTTTAATATCAATACC  963

Query  6768  AACTGGTACAAAAATCTTTAATTGGCTTTTTACATATCTCTCCAATCCACCATTATTACA  6827
              ** ********* * ***** *** * * ******* *    * *     * * **
Sbjct   964  TACCGGTACAAAAGTATTTAACTGGATATGTACATATATGAGTAGT-----AAT-TTTGG  1017

Query  6828  CCTTAGAATTACTTCTGTCTTCCTCTCACATCTCTTTTTATTAATGTTTACGGTAGGTGG  6887
               * * *   *  ***   **  * ***    * *** ***  *  *****  * ** **
Sbjct  1018  TATGATACACAGCTCTTCATTATTGTCATTATTATTTATATGTACATTTACATTTGGAGG  1077

Query  6888  GTCAACAGGAATAATTCTTGGAAATGGTGCAGTGGATCTAGGATTACATGATACATATTA  6947
               * ** *** * **  * ** **** ***  * *** *** ********* ********
Sbjct  1078  TACTACTGGAGTTATATTAGGTAATGCTGCCATTGATGTAGCATTACATGACACATATTA  1137

Query  6948  TGTTGTAGCACATTTTCATTTTGTTCTTTCTTTAGGAGCTATAATTGCTATCTTCTCTGG  7007
             **** * ** ***** ******** ** **  * ** ** ** ****   * **  *
Sbjct  1138  TGTTATTGCTCATTTCCATTTTGTACTATCAATTGGTGCAATTATTGGATTATTTACAAC  1197

Query  7008  AATAATCTTGAATGGAGAAAAGATTGTTGCTACTAAGAATTTATTACTTTCATCCTCATG  7067
               ***       *  *** **  *  *** *   ** ** ***        ** **  **
Sbjct  1198  TGTAAGTGCATTTCAAGATAATTTCTTTGGT---AAAAACTTA-CGTGAAAATTCTATTG  1253

Query  7068  TACACTCTCTCTTTATCATTTACATTTAATATTTATTGGTATTCTTCTTACCTTTTCCCC  7127
             ** *  ** *  * *  ** *  ****    *** * *** *  *  * ** ****  **
Sbjct  1254  TA-ATACTATGGTCA--ATGT-TATTT----TTTGTAGGTGTAATATTAACATTTTTACC  1305

Query  7128  AATGCATTTCTTAGGATTTAATCTTATGCCAAGAAGAATCCCATCCTTTCCAGATTCTTT  7187
              ******** ************ * ***** *** * ** **    * ******  ****
Sbjct  1306  TATGCATTTTTTAGGATTTAATGTAATGCCTAGACGTATTCCTGATTATCCAGACGCTTT  1365

Query  7188  TCATTCCTGGAATTCCCTGTCATCTATTGGATCAGGAATAACTTTCCTATCTTTTTCTAC  7247
               **   ******    * *  ******** ***  *** *****  * *  *** ***
Sbjct  1366  AAATGGATGGAATATGATTTGTTCTATTGGGTCAACAATGACTTT-ATTTGGTTTACTAA  1424

Query  7248  T  7248
             *
Sbjct  1425  T  1425


1st Nucleotide Sequence
  File Name        : S_minutum_mtDNA-v1.seq
  Sequence Size    : 326535

2nd Nucleotide Sequence
  File Name        : cox3_lcl|M76611.1[725-1487]c
  Sequence Size    : 763

Query Range: 186587 - 187332
Sbjct Range: 1 - 752
Identity: 405 / 771 (52%)
Similarity: 405 / 771 (52%)
Gaps: 44 / 771 (5%)
Strand: Plus / Plus

Query  186587  ATTTATCTT-TACAG-GATTTAATATTTATTGTTGGACTGGAATTTACTTCTCATAGAAT  186644
               ****** ** *  **  *****  *  ***    * **    * ** * * ** *  * *
Sbjct       1  ATTTATTTTATTTAGTAATTTATCAAATATAAAAGCACATCTAGTTTCATATCCTGCATT  60

Query  186645  TATTTTCTTATAT--TATTTCATCTTAAT-TTTGGATTAATTTATAGTTTTCATATTT-G  186700
                *  *  ******  **  ** *   ***  **   *  *   *** * ** * ****
Sbjct      61  AACATCATTATATGGTACATCTTTAAAATACTTTTCTGTAGGGATATTATTTACATTTAA  120
```

```
Query  186701  GTCTAGAGATTTATTAA-----GAGAATTC---ACTAAAAAATATGAAGTCTTATTAATA  186752
                 *** *    ******     *   ****    * * *** * *  * ***   *  *
Sbjct     121  CCCTATAATCCTATTAATATTTGTATATTCTATTCGAGAAAGTTTTTATTCTGTATTTTC  180

Query  186753  ATCTTTTTTCTTCT-TTTTGGGGGTTTTC-TAGTTTCTGAAGCTCTATTATTTGTATCCT  186810
               *** ***  *****  * **     * ** ** * ** ****** *******  *  * *
Sbjct     181  ATC-TTTAACTTCTGGTATGTTATCTATCATAATATCAGAAGCTTTATTATTCTTTACAT  239

Query  186811  TCTTTTGGACATCTTTTCATTTATTATCTTCTCCAACGGCTGGGATGTGGCTGTGGGAAG  186870
                 ******      ** *****    *  ** ** *   *    * **   **  ** *
Sbjct     240  ATTTTTGGGGTATATTACATTTTAGTTTATCACC-ATATCCATTAAGT-AATGAAGGTAT  297

Query  186871  CTTTCTATCTGGAGGATCCTTGTGAATTAACTTTTGCTAATACACTTCTTTTATCAAATG  186930
                 *  *  **  *  *    ***    ***  **  * * **** ** * *** * * **
Sbjct     298  TATCATTACTTCATCAAGAATGT----TAATCTTAACAATTACATTTATATTAGCTAGTG  353

Query  186931  C-TGCTATAT-----CTTTAGGAAATGCTTTTATTAATTTAGAAATTTCATCAGAATATA  186984
               * *  * ***     * *    * * *  *****  *   ** ***    *  *** **
Sbjct     354  CATCATGTATGACTGCATGTTTACAAGTATTTATAGAAAAAGGAATGAGTTTTGAA-AT-  411

Query  186985  TTATTTTCTTCACTTTATGGTCATTCTTTTTGTCTTCTCTTTTTATTAGTTTGCAGATTA  187044
                * *  * **   * ***  * ** *** **         ****     * * ** * **
Sbjct     412  CTCTAGTATTATTTGTATAAT-ATACTTATTAGGAGAATGTTTTGCATCTCTACAAACTA  470

Query  187045  AAGAAT-TTCGCATTCTCGCATTATCA-ATTAATGATTCACTTTATAGTTCTCTTTTCTT  187102
                *** * **  **** *  ** ***** ** ****** *  * **** * *  * ** *
Sbjct     471  CAGAGTATTTACATT-TATCA-TATCATATAAATGATACTGTATATACTACATTATTTTA  528

Query  187103  TTTTCTTACAGGATTACATTTCTTTCATCTATCTATTGGTCTTATTCTTCTAATTTATTT  187162
               ** * **************** * **** **   ** *** ***** ** * * * ** *
Sbjct     529  TTGTGTTACAGGATTACATTTTTCTCATGTAGTAATAGGT-TTATTATTATTAATAATAT  587

Query  187163  CTATTATGTGGGTTTCTTTGTTTACTTTATGCTTTCATGGACTTCTTTTAATAAAATATG  187222
                  ****   *  *  *     **  * ** ***  *  **  *  *** ***** * *
Sbjct     588  ACTTTAT-AAGAATAATAGAAATATATGATACTTCTACCGA-ATGGTTT-ATAAATTCT-  643

Query  187223  ATTGGAAATTATTGGTATTTTCTAGGAGTTTCTCGGGTAATAATCTCTTGCAATCCTATC  187282
                ** *  **      **** * **    *  * * * * * * * *    *   **  *  *
Sbjct     644  -TTCGGTATATCATATATTGT-TATACCTCACACTGATCAAATTACAATTTTATATTGGC  701

Query  187283  -TTCTGTTTCCCCTTTCTGGCCTATGATCACATTAATAATCCCTTAGAAAT  187332
                ** ****       *****    * **    **  ** **  **   ***
Sbjct     702  ATTTTGTTGAAATAATCTGGTTATTTATAGAGTTCTTATTCTATTCAGAAT  752



1st Nucleotide Sequence
  File Name        : S_minutum_mtDNA-v1.seq
  Sequence Size    : 326535

2nd Nucleotide Sequence
  File Name        : cob_lcl|M76611.1[3480-4624]
  Sequence Size    : 1145

Query Range: 197602 - 198718
Sbjct Range: 10 - 1131
Identity: 688 / 1131 (60%)
Similarity: 688 / 1131 (60%)
Gaps: 23 / 1131 (2%)
Strand: Plus / Plus

Query  197602  TTAAAACTCTTATATTTTTTATTACTAATGAAATCTCATTTACAATCATATCCTTGTCCT  197661
               ** *    *** ** * * *     ** * *** * ** ***  *   ** ** *****
Sbjct      10  TTTATGAACTTTTACTCTATTAATTTAGTTAAAGCACACTTAATAAATTACCCATGTCCA  69

Query  197662  CTGATCATAAATTATTTTTGGAATCTTGGTTTTTTATTAGGGATTACTATTTTATTACAA  197721
                *** ****** * *** ******   ** **  * ***** ** *   ****  * ***
Sbjct      70  TTGAACATAAACTTTTTATGGAATTACGGATTCCTTTTAGGAATAATATTTTTTATTCAA  129
```

```
Query  197722  ATTATATCTGGAATCTTCTTAGGTTTACATTATACATCAGATATTAATTCAGCATATTTT  197781
               ****** * **  * ** ****     *  ****** ***** **   *  ******* *
Sbjct     130  ATTATAACAGGTGTATTTTTAGCAAGTCGATATACACCAGATGTTTCATATGCATATTAT  189

Query  197782  AGTATTTTCTTTATTATTAGAGAAATATATTATGGATGGTGTTTACGTTATCTTCATTCT  197841
               *****       *** * ****** ***    ************  * **  * **  *
Sbjct     190  AGTATACAACACATTTTAAGAGAATTATGGAGTGGATGGTGTTTTAGATACATGCACGCA  249

Query  197842  AATGGTTCATCATTTGTCTTTCTTTTTATATTTCTACATCTTGGAAGAGCTATATCTTAT  197901
               *  *** * **  **** *** * ** * ** *** *** **  *****   **  ***
Sbjct     250  ACAGGTGCTTCTCTTGTATTTTTATTAACATATCTTCATATTTTAAGAGGATTAAATTA-  308

Query  197902  GGTTCATATTTTTATAATCCAAATACTTGGTTTTCTGGAATTATTATTATTTTCTTCTTG  197961
                  ****** * ***   ***    * *** * ****** * *** *  ** *  * **
Sbjct     309  --CTCATATATGTATTTACCATTATCATGGATATCTGGATTGATTTTATTTATGATATTT  366

Query  197962  ATGGGAACAGCATTTATGGGTTATGTGTTACCTTTAGGACAAATGAGTTTATGGGGGGTT  198021
               ** * *** ** **  * ******** ***** *  ** **********  ***** *
Sbjct     367  ATTGTAACTGCTTTCGTTGGTTATGTCTTACCATGGGGTCAAATGAGTTATTGGGGTGCA  426

Query  198022  ACAGTAATTACAAATTTATTATCTGCATTTCCATCTTTAATAGAATGGCTTTGTGGAGGA  198081
               ** ******** ** ** *****  *  *****     * **   *** * *********
Sbjct     427  ACTGTAATTACTAACTTGTTATCCTCTATTCCAGTAGCAGTAATTTGGATATGTGGAGGA  486

Query  198082  CATTACATTTACAATCCTACATTTAAGAGGTTCTTTGTCTTTCATTTTCTATTTCCATTT  198141
                **    *     ******** * **  * ** *****  * ****** * ** ******
Sbjct     487  TATACTGTGAGTGATCCTACAATAAAACGATTTTTTGTACTACATTTTATCTTACCATTT  546

Query  198142  CTTCTTTGTGGTTTTCTTCTTTATCATATTTTTAATCTACATTTTCTATCTTCTAATAAT  198201
                **   *   ** ** *  **   ***** ***    ******* *    *   *  ***
Sbjct     547  ATTGGATTATGTATTGTATTTATACATATATTTTTCTTACATTTACATGGTAGCACAAAT  606

Query  198202  CCTTTAAGGAATTCCACTAATAATAAAATAGCATTTTTGCCTTTCATTATTAGTAAAGAT  198261
               ****** ** **   **       ****** * ****  **      *  * ***   ***
Sbjct     607  CCTTTAGGGTATGATACAGCATTAAAAATACCCTTTTATCCAAATCTATTAAGTCTTGAT  666

Query  198262  TTCTATGGAATGATATTAATTTCCTA-TCTATATCTTCTTCAGATTCATTTTGGATTCTC  198320
                *  * *** * * ** *  **   * * *** ***  * ** * *   ****** *
Sbjct     667  GTTAAAGGATTTA-ATAATGTTATAATTTTATTTCTAATACAAAGTTTATTTGGAATTAT  725

Query  198321  TTCTTTCTCACATCCAGATAATGCATTGGAAGCTTGTGGATTACTTACACCTTTACACAT  198380
                 **** ******** ********  * * **    *  **   **** ** *  ** **
Sbjct     726  ACCTTTATCACATCCTGATAATGCTATCGTAGTAAATACATATGTTACTCCATCTCAAAT  785

Query  198381  TGTACCTGAATGGTATTTTCTATGCCAATATGCTATGTTAAAAGCTGTACCCAATAAAAA  198440
               *************** ******      ***** ********* **** ** * ****
Sbjct     786  TGTACCTGAATGGTACTTTCTACCATTTTATGCAATGTTAAAAACTGTTCCAAGTAAACC  845

Query  198441  TGCAGG---ATTCATTGTCTTACTA--ACTTCTA-TCTTTGTATTATTTTATTTTATGAG  198494
                ** **   * * ***** *** **  * * * * * ** * *****          **
Sbjct     846  AGCTGGTTTAGTAATTGTATTATTATCATTACAATTATTATTCTTATTAGCAGAACAAAG  905

Query  198495  AAGTCTTTCAA-TATCTTTCTATTTTATCGTGTGGGTAAGCTCTAGATTTAATAGTTTCT  198553
               **** *  *** ***  *** * ****    **    *  *  *****   * ** * * *
Sbjct     906  AAGTTTAACAACTATAATTC-AATTTAAAATGATTTTTGGTGCTAGA--GATTATTCTGT  962

Query  198554  TTGTAATTCTTTGGTTTTTAGTTTTCA---TATCCTTAATT-TGGATAGGTGGTCAATTT  198609
               *  * *** * ****** * ** * **   *** *** *** ***** **  *******
Sbjct     963  TCCT-ATTATATGGTTTAT-GTGTGCATTCTATGCTTTATTATGGATTGGATGTCAATTA  1020

Query  198610  CCTGTCGACAACTTTCTATCTTATGGTCGTATCTTGACATTA-TATTATTAT-TATTTTC  198667
               **     ** *  **  * *  ********  * ** *   ** **** ** * ** *
Sbjct    1021  CCACAAGATATATTCATTTTATATGGTCGATTATTTATTGTATTATTTTTCTGTAGTGGT  1080

Query  198668  TTATCTGTATCTTATTATCTTAGGGGGGCTTTGGTGGGTTCCACTGCGCTA  198718
               **** ****  *  * **  ***  *  *       * ** **   * * *
Sbjct    1081  TTATTTGTACTTGTTCATTATAGACGAACACATTATGATTACAGCTCCCAA  1131
```

1st Nucleotide Sequence
  File Name   : S_minutum_mtDNA-v1.seq

```
  Sequence Size    : 326535

2nd Nucleotide Sequence
  File Name        : SSUA_lcl|M76611.1[2023-1916]c
  Sequence Size    : 108

Query Range: 177279 - 177354
Sbjct Range: 28 - 106
Identity: 57 / 80 (71%)
Similarity: 57 / 80 (71%)
Gaps: 5 / 80 (6%)
Strand: Plus / Plus

Query  177279  ATCTAGTCCCAGCTGCAGCGGTAAGACTATTGAAAAGACGAGTCTT----ATCCAGACCA  177334
               **  *********  ******* * *** * *** ** ***** **    *****   **
Sbjct      28  ATACAGTCCCAGCGACAGCGGTTATACTTTGGAAGAGTCGAGTATTATCCATCCATGTCA  87

Query  177335  GGCGTTAACAGTCCTTAGGT  177354
               ******** ** * ** * *
Sbjct      88  GGCGTTAAAAG-CGTTCGTT  106


1st Nucleotide Sequence
  File Name        : S_minutum_mtDNA-v1.seq
  Sequence Size    : 326535

2nd Nucleotide Sequence
  File Name        : SSUB_lcl|M76611.1[505-390]c
  Sequence Size    : 116

Query Range: 236311 - 236394
Sbjct Range: 18 - 103
Identity: 48 / 86 (55%)
Similarity: 48 / 86 (55%)
Gaps: 2 / 86 (2%)
Strand: Plus / Plus

Query  236311  AGGTACTAACAATAAGAAAGAAATGGTATAAAATATAAAAATA-TAATTAATTATTACAG  236369
               * ** *  *** * * **  ** *** **   * *   *** * **       *  ***
Sbjct      18  ACGTCCATACAGTTATAAGCAAGTGGAATGTTAGAAGCAAACACTAGCGGTGGAACACAT  77

Query  236370  CTCTTC-CATCCTAGTAAACACTATA  236394
                  ***   *  **************
Sbjct      78  TGTTTCATTTGATAGTAAACACTATA  103


1st Nucleotide Sequence
  File Name        : S_minutum_mtDNA-v1.seq
  Sequence Size    : 326535

2nd Nucleotide Sequence
  File Name        : SSUD_lcl|M76611.1[5446-5379]c
  Sequence Size    : 68

Query Range: 176902 - 176959
Sbjct Range: 3 - 65
Identity: 37 / 63 (58%)
Similarity: 37 / 63 (58%)
Gaps: 5 / 63 (7%)
Strand: Plus / Minus

Query  176902  ATCCTAGGTGAGCCGACATACAATATTTCAT-----TTATAATCTGTTTCTACTTCCTAC  176956
               ** ***   **  * ** * **  * *** *     ***  * *  ***********  *
Sbjct      65  ATGCTATTGGATTCAACGTCCAGGACTTCCTGACGCTTAATAACGATTTCTACTTCCAGC  6

Query  176957  GGC  176959
                **
Sbjct       5  AGC  3


1st Nucleotide Sequence
```

```
  File Name        : S_minutum_mtDNA-v1.seq
  Sequence Size    : 326535

2nd Nucleotide Sequence
  File Name        : SSUE_lcl|M76611.1[1638-1680]
  Sequence Size    : 43

Query Range: 221699 - 221724
Sbjct Range: 9 - 34
Identity: 19 / 26 (73%)
Similarity: 19 / 26 (73%)
Gaps: 0 / 26 (0%)
Strand: Plus / Plus

Query  221699  ATTATTACCTTGTAACCCTGGCTCGT  221724
               *** ** *******  *   ******
Sbjct       9  ATTGTTGCCTTGTACACACCGCTCGT  34


1st Nucleotide Sequence
  File Name        : S_minutum_mtDNA-v1.seq
  Sequence Size    : 326535

2nd Nucleotide Sequence
  File Name        : SSUF_lcl|M76611.1[5507-5447]c
  Sequence Size    : 61

Query Range: 170409 - 170456
Sbjct Range: 14 - 59
Identity: 31 / 48 (64%)
Similarity: 31 / 48 (64%)
Gaps: 2 / 48 (4%)
Strand: Plus / Plus

Query  170409  GAATCCCTTCTAAATTCCTTGAAAGTGAACCTAGAAAGCTGAACCAGA  170456
               ***  * *   *   *  **** ****** ** *  ********** *
Sbjct      14  GAAGTCGTAACATGGTAGTTGACAGTGAA-CTTG-TAGCTGAACCAAA  59


1st Nucleotide Sequence
  File Name        : S_minutum_mtDNA-v1.seq
  Sequence Size    : 326535

2nd Nucleotide Sequence
  File Name        : LSUA_lcl|M76611.1[5201-5026]c
  Sequence Size    : 176

Query Range: 105339 - 105493
Sbjct Range: 1 - 157
Identity: 89 / 158 (56%)
Similarity: 89 / 158 (56%)
Gaps: 4 / 158 (2%)
Strand: Plus / Minus

Query  105339  GATTTTACAGATAACCGTCTATTACAAAGAATTATTGACCATTAACTGCCAAGGAACAAA  105398
               *****  **** **********    * *  * ******* *****  * **   ** **
Sbjct     157  GATTTCGCAGAAAACCGTCTATATTCATGTTTGATTGACCTTTAACCACTAA-TTACGAA  99

Query  105399  TCATCCAAGAAGCATTCTATCTTTCATGGACATTACTGGT-TAATATTG-TCGGGTCTTA  105456
               ** ********   **  *   * ** **      **  * *  *  ** ** *   ***
Sbjct      98  TCTTCCAAGAATATTTTAAGAGTCCAAGGTTCGGTCTATTATTTTCCTGTTCTGTAATTA  39

Query  105457  CA-CTCATTATTCCTACCGATGGCTCCCATCACATTAA  105493
                * * ***  **  **      *    ***  *  ***
Sbjct      38  GATCACATGTTTTATAGTTCATGGAGACATGGCTATAA  1


1st Nucleotide Sequence
  File Name        : S_minutum_mtDNA-v1.seq
  Sequence Size    : 326535
```

```
2nd Nucleotide Sequence
  File Name        : LSUB_lcl|M76611.1[4618-4586]c
  Sequence Size    : 33

Query Range: 38813 - 38831
Sbjct Range: 9 - 27
Identity: 17 / 19 (89%)
Similarity: 17 / 19 (89%)
Gaps: 0 / 19 (0%)
Strand: Plus / Minus

Query  38813  TTATGATTACACCTACCAA  38831
              *********** ** ****
Sbjct     27  TTATGATTACAGCTCCCAA  9


1st Nucleotide Sequence
  File Name        : S_minutum_mtDNA-v1.seq
  Sequence Size    : 326535

2nd Nucleotide Sequence
  File Name        : LSUC_lcl|M76611.1[225-204]c
  Sequence Size    : 22

Query Range: 73563 - 73580
Sbjct Range: 4 - 21
Identity: 17 / 18 (94%)
Similarity: 17 / 18 (94%)
Gaps: 0 / 18 (0%)
Strand: Plus / Minus

Query  73563  AGAGCTAGGACGCTATCA  73580
              ******* **********
Sbjct     21  AGAGCTATGACGCTATCA  4



1st Nucleotide Sequence
  File Name        : S_minutum_mtDNA-v1.seq
  Sequence Size    : 326535

2nd Nucleotide Sequence
  File Name        : LSUD_lcl|M76611.1[5854-5772]c
  Sequence Size    : 83

Query Range: 222761 - 222836
Sbjct Range: 2 - 77
Identity: 55 / 76 (72%)
Similarity: 55 / 76 (72%)
Gaps: 0 / 76 (0%)
Strand: Plus / Plus

Query  222761  TGGAAGCGTCTGTAAGGTTGGGACACAAGACCCTGAGAATTTGGATGAAGAAGTCAAGCC  222820
               *******************   ******* * **** ****  ****** *  *****
Sbjct       2  TGGAAGCGTCTGTAAGGTTACAACACAAGTCACTGATAATTCTGATGAATATTTCAAGTT  61

Query  222821  TTTGGTAACAGTCTGG  222836
                 **  * * * * **
Sbjct      62  ACTGACATCTGCCCGG  77


1st Nucleotide Sequence
  File Name        : S_minutum_mtDNA-v1.seq
  Sequence Size    : 326535

2nd Nucleotide Sequence
  File Name        : LSUE_lcl|M76611.1[5771-5577]c
  Sequence Size    : 195

Query Range: 193381 - 193573
```

```
Sbjct Range: 3 - 192
Identity: 149 / 195 (76%)
Similarity: 149 / 195 (76%)
Gaps: 7 / 195 (3%)
Strand: Plus / Plus

Query  193381  ACACACGGCGGCTGTAAGGTTAGGAAAGGTCCTAAGGTAGCAAAATTCCTTGACAGGTAA  193440
               * * *********** *  ***  ** ************************* * *****
Sbjct       3  ATAAACGGCGGCTGT-ATTTA--AACGGTCCTAAGGTAGCAAAATTCCTTGTCGGGTAA  59

Query  193441  GTTCCGTCCAGCATGAGCGGTGTAAGGACTTCCTCACTGTCACTAGCCT-CGTCT-CTCA  193498
                 ******* ****** ******** ******* ** **** ****  *  * ** ** *
Sbjct      60  TCTCCGTCCTGCATGAACGGTGTAACGACTTCCCCATTGTCGCTAGTGTGAGACTCCTAA  119

Query  193499  GAAATTGAGTCATCCTTGATTACGAGGAAGCCAACGGCCTGACAATAAGACCCTGAGCAC  193558
                **** ** * **** *** ** * **** *  ****** ***  ***************
Sbjct     120  TAAATAGAATTATCCATGAATATGTGGAATCATACGGCCCGACGGTAAGACCCTGAGCAC  179

Query  193559  CAGTTTTCTCTCTTA  193573
               *  **  **  * **
Sbjct     180  C--TTAACTTCCCTA  192


1st Nucleotide Sequence
  File Name        : S_minutum_mtDNA-v1.seq
  Sequence Size    : 326535

2nd Nucleotide Sequence
  File Name        : LSUF_lcl|M76611.1[1516-1630]
  Sequence Size    : 115


Query Range: 279828 - 279907
Sbjct Range: 20 - 99
Identity: 55 / 80 (68%)
Similarity: 55 / 80 (68%)
Gaps: 0 / 80 (0%)
Strand: Plus / Plus

Query  279828  GGCTCGCCAAGGATAACAGGTTCTTGAATCCTAAGAGCTCCTACAAAAGGATTCTGATGG  279887
               ** *****  ************ * * **    *******  *       ** **  ***
Sbjct      20  GGTTCGCCGGGGATAACAGGTTATAGTATATATAGAGCTCTAATCTTTATATACTATTGG  79

Query  279888  CACCTTCATGTCGGTTCATC  279907
               ***** *******  *****
Sbjct      80  CACCTCCATGTCGTCTCATC  99



1st Nucleotide Sequence
  File Name        : S_minutum_mtDNA-v1.seq
  Sequence Size    : 326535

2nd Nucleotide Sequence
  File Name        : LSUG_lcl|M76611.1[389-283]c
  Sequence Size    : 107

  Unit Size to Compare = 6
  Pick up Location     = 10

Query Range: 278801 - 278900
Sbjct Range: 10 - 107
Identity: 74 / 100 (74%)
Similarity: 74 / 100 (74%)
Gaps: 2 / 100 (2%)
Strand: Plus / Minus

Query  278801  TAAAATGAAGACCATGAGGTAGAAACTGCCGGTGGACGCTATTAACCCAGCTCACGGATC  278860
               ***** *  *   **  ** * ********    **** * *************** ***
Sbjct     107  TAAAACG--GTAGATAGGGAACAAACTGCCTCAAGACGTTCTTAACCCAGCTCACGCATC  50
```

```
Query  278861  GGTTCTAAGGGTGAACTCTCCTTCCCTAGGAATCTTCTCC  278900
               * ****** *********** ****   **** *** * *
Sbjct      49  GCTTCTAACGGTGAACTCTCATTCCAATGGAACCTTGTTC  10


1st Nucleotide Sequence
  File Name        : S_minutum_mtDNA-v1.seq
  Sequence Size    : 326535

2nd Nucleotide Sequence
  File Name        : RNA1_lcl|M76611.1[593-506]c
  Sequence Size    : 88

Query Range: 317063 - 317147
Sbjct Range: 4 - 88
Identity: 54 / 88 (61%)
Similarity: 54 / 88 (61%)
Gaps: 6 / 88 (6%)
Strand: Plus / Plus

Query  317063  TCATATTTACTATG--GA-TTCATTATGGAAAAACATGGAAAACATATAAAAATTAATTG  317119
               ****** *******  ** ** * **  ** ***  * ** *  **  *   **  ** *
Sbjct       4  TCATATATACTATGCTGACTTGAGTAATGATAAA--TTGATAGTAT-CAGCTATCCATAG  60

Query  317120  ATTTTTGAGTGATTTTTGAGTGATTATT  317147
                *  **** *   ******  * * ***
Sbjct      61  TTAATTGATTCCGTTTTGACCGGTCATT  88


1st Nucleotide Sequence
  File Name        : S_minutum_mtDNA-v1.seq
  Sequence Size    : 326535

2nd Nucleotide Sequence
  File Name        : RNA2_lcl|M76611.1[1698-1763]
  Sequence Size    : 66

Query Range: 60688 - 60729
Sbjct Range: 21 - 62
Identity: 26 / 42 (61%)
Similarity: 26 / 42 (61%)
Gaps: 0 / 42 (0%)
Strand: Plus / Plus

Query  60688  TAAAGTCATGATGGAGCTGAAAGGAAAGGTAAACTGATGGCC  60729
              ** ***   ******       **********  * * * **
Sbjct     21  TAGAGTTGAGATGGAAACAGCCGGAAAGGTAATTTTACGCCC  62


1st Nucleotide Sequence
  File Name        : S_minutum_mtDNA-v1.seq
  Sequence Size    : 326535

2nd Nucleotide Sequence
  File Name        : RNA3_lcl|M76611.1[1830-1910]
  Sequence Size    : 81

Query Range: 34514 - 34593
Sbjct Range: 2 - 81
Identity: 45 / 81 (55%)
Similarity: 45 / 81 (55%)
Gaps: 2 / 81 (2%)
Strand: Plus / Minus

Query  34514  TGTAGGCTGGGACCATTCCTTTATTATCCTTT-CTGGTACCTTCAATGGCTCATCATTCC  34572
              *******  *     ******** * *  *** **   * *** * * * **   * * *
Sbjct     81  TGTAGGCCAGTCGAGTTCCTTTAATGTAGTTTCCTCACAGCTTTATTCGGTCCAAAGTAC  22

Query  34573  TTAATAAATTTCTAGTTTAAT  34593
                 **    ** **   ****
Sbjct     21  GCGAT-CTCTTGTATGGTAAT  2
```

```
1st Nucleotide Sequence
  File Name        : S_minutum_mtDNA-v1.seq
  Sequence Size    : 326535

2nd Nucleotide Sequence
  File Name        : RNA4_lcl|M76611.1[4625-4696]
  Sequence Size    : 72

Query Range: 220439 - 220506
Sbjct Range: 2 - 68
Identity: 39 / 68 (57%)
Similarity: 39 / 68 (57%)
Gaps: 1 / 68 (1%)
Strand: Plus / Minus

Query  220439  ATGAGAGCAAAACAGCTAGATTTGGTTAGGAAAATACTCAAGAAATAATATTAAATCTAG  220498
               ** * * *   ** *   ****** *  *   ******* ******   **   ***
Sbjct      68  ATTATAACCTTACGGTCTGATTTGTTCCGCTCAATACTC-AGAAATGTCATCTTATCACA  10

Query  220499  CTGGTGTA  220506
                *  ****
Sbjct       9  ATCTTGTA  2


1st Nucleotide Sequence
  File Name        : S_minutum_mtDNA-v1.seq
  Sequence Size    : 326535

2nd Nucleotide Sequence
  File Name        : RNA5_lcl|M76611.1[4716-4802]
  Sequence Size    : 87

Query Range: 138204 - 138280
Sbjct Range: 2 - 81
Identity: 48 / 80 (60%)
Similarity: 48 / 80 (60%)
Gaps: 3 / 80 (3%)
Strand: Plus / Plus

Query  138204  ATTATAGTTTCTTTTGTTATGGATGAATTAAT-TAT-TAGTATAAATTTCTTTGTCATTC  138261
               * ******* *  * * * * **** **   *  ** **   ** *** *  * *** **
Sbjct       2  ACTATAGTTACCATAGCTGTAGATGGATGCTTCGATATATAGTATATTACAGTATCAATC  61

Query  138262  AGTTTTA-ACGGTTAGTCCC  138280
                * **** * * * ** * *
Sbjct      62  GGATTTACATGCTCAGCCGC  81


1st Nucleotide Sequence
  File Name        : S_minutum_mtDNA-v1.seq
  Sequence Size    : 326535

2nd Nucleotide Sequence
  File Name        : RNA6_lcl|M76611.1[4808-4865]
  Sequence Size    : 58

  Unit Size to Compare = 6
  Pick up Location     = 10

Query Range: 14596 - 14626
Sbjct Range: 2 - 34
Identity: 27 / 33 (81%)
Similarity: 27 / 33 (81%)
Gaps: 2 / 33 (6%)
Strand: Plus / Minus

Query  14596  GTCTTGCTAAC-GCTTCTAAGG-AATATTATTG  14626
              *********** **** ** ** **** *** *
Sbjct     34  GTCTTGCTAACGGCTTGTACGGTAATAATATCG  2
```

```
1st Nucleotide Sequence
  File Name        : S_minutum_mtDNA-v1.seq
  Sequence Size    : 326535

2nd Nucleotide Sequence
  File Name        : RNA7_lcl|M76611.1[5283-5202]c
  Sequence Size    : 82

Query Range: 56199 - 56266
Sbjct Range: 14 - 81
Identity: 53 / 69 (76%)
Similarity: 53 / 69 (76%)
Gaps: 2 / 69 (2%)
Strand: Plus / Plus

Query  56199  AGAATCCTAAAGGTAACAAAGAATCCTGGAAATCGAGGGAGATGTCGTAAGTTGTCTTTA  56258
              ********    *****  *  ** ************ *****  * * ******** **
Sbjct     14  AGAATCCTCTTAGTAACTCAACAT-CTGGAAATCGAGAGAGATTCCATTAGTTGTCTCTA  72

Query  56259  TG-ATAGTG  56266
              ** ******
Sbjct     73  TGAATAGTG  81


1st Nucleotide Sequence
  File Name        : S_minutum_mtDNA-v1.seq
  Sequence Size    : 326535

2nd Nucleotide Sequence
  File Name        : RNA8_lcl|M76611.1[5954-5855]c
  Sequence Size    : 100

Query Range: 106227 - 106279
Sbjct Range: 48 - 100
Identity: 30 / 53 (56%)
Similarity: 30 / 53 (56%)
Gaps: 0 / 53 (0%)
Strand: Plus / Minus

Query  106227  TCATAAAAGCTCATATAAAATCTGATGTAGGAAGCCTTTGAATCCAATCCATT  106279
               * **  **  ** ******       *  **   **   * ********** *
Sbjct     100  TAATTTAACTTCTTATAAATGGAAGCGCCGGTTTCCCGGGTATCCAATCCAGT  48


1st Nucleotide Sequence
  File Name        : S_minutum_mtDNA-v1.seq
  Sequence Size    : 326535

2nd Nucleotide Sequence
  File Name        : RNA9_lcl|M76611.1[72-125]
  Sequence Size    : 54

Query Range: 281819 - 281866
Sbjct Range: 1 - 50
Identity: 34 / 50 (68%)
Similarity: 34 / 50 (68%)
Gaps: 2 / 50 (4%)
Strand: Plus / Plus

Query  281819  TAATAAGGCCAGCCCACACCATACAAATACAAT--AAATTTTGTGTTTTA  281866
               * * * * ****** ******* *** *  * *   **** * *** ***
Sbjct       1  TTAAATGCCCAGCCAACACCATCCAATTTGATTGGGAATTATCTGTGTTA  50



1st Nucleotide Sequence
  File Name        : S_minutum_mtDNA-v1.seq
  Sequence Size    : 326535
```

```
2nd Nucleotide Sequence
  File Name        : RNA10_lcl|M76611.1[724-625]c
  Sequence Size    : 100

Query Range: 217165 - 217255
Sbjct Range: 3 - 94
Identity: 59 / 92 (64%)
Similarity: 59 / 92 (64%)
Gaps: 1 / 92 (1%)
Strand: Plus / Plus

Query  217165  TTTCCTTTCCCATATACTTCTAT-TATCTTCTAATCATTAATTAAGTCATGAGATTGATG  217223
               * **** *  ** ***  * ***  **  *   *  * **   ** *      ** * *
Sbjct       3  TGTCCTGTTTCAAATATATATATGAATAATTGTACGAATAGACAATTGTGTTCATAGCTA  62

Query  217224  GTGTACGAAAGGAAAAGGAAAGGTTAACCGCT  217255
               * ***** ************************
Sbjct      63  GAGTACGTAAGGAAAAGGAAAGGTTAACCGCT  94


1st Nucleotide Sequence
  File Name        : S_minutum_mtDNA-v1.seq
  Sequence Size    : 326535

2nd Nucleotide Sequence
  File Name        : RNA11_lcl|M76611.1[5340-5284]c
  Sequence Size    : 57

Query Range: 73434 - 73479
Sbjct Range: 8 - 53
Identity: 29 / 46 (63%)
Similarity: 29 / 46 (63%)
Gaps: 0 / 46 (0%)
Strand: Plus / Minus

Query  73434  TTCTTCCAAGATAAGTTTCTACTTTCATGTCCTAGTGTACAAGCAT  73479
              * ******  *  **  * **** ** *** ***   * * ** **
Sbjct     53  TACTTCCACTACCAGAATATACTCTCCTGTTCTAAAATTCTAGGAT  8


1st Nucleotide Sequence
  File Name        : S_minutum_mtDNA-v1.seq
  Sequence Size    : 326535

2nd Nucleotide Sequence
  File Name        : RNA12_lcl|M76611.1[4887-4945]
  Sequence Size    : 59

Query Range: 191852 - 191892
Sbjct Range: 15 - 54
Identity: 30 / 41 (73%)
Similarity: 30 / 41 (73%)
Gaps: 1 / 41 (2%)
Strand: Plus / Plus

Query  191852  TAGGATTTATGGGTTTATTTGTATAAGAATTTCATGGTTTT  191892
               ****** ******  ********  * *  ** ** * ***
Sbjct      15  TAGGATGTATGGG-ATATTTGTAGTACACCTTGATTGGTTT  54



1st Nucleotide Sequence
  File Name        : S_minutum_mtDNA-v1.seq
  Sequence Size    : 326535

2nd Nucleotide Sequence
  File Name        : RNA13_lcl|M76611.1[5025-4996]c
  Sequence Size    : 30

Query Range: 256591 - 256614
```

```
Sbjct Range: 1 - 24
Identity: 17 / 24 (70%)
Similarity: 17 / 24 (70%)
Gaps: 0 / 24 (0%)
Strand: Plus / Minus

Query  256591  CTGCCCCCGTGTTTAACCTTCCCA  256614
               *** *  * **  *** *******
Sbjct      24  CTGACTTCCTGGCTAAACTTCCCA  1


1st Nucleotide Sequence
  File Name        : S_minutum_mtDNA-v1.seq
  Sequence Size    : 326535

2nd Nucleotide Sequence
  File Name        : RNA14_lcl|M76611.1[5546-5508]c
  Sequence Size    : 39

Query Range: 31151 - 31177
Sbjct Range: 1 - 27
Identity: 21 / 27 (77%)
Similarity: 21 / 27 (77%)
Gaps: 0 / 27 (0%)
Strand: Plus / Plus

Query  31151  TATGATTGAAACCTTCATGAACGATTC  31177
              ** *  ********** *** *** **
Sbjct      1  TAAGGATGAAACCTTCCTGATCGACTC  27


1st Nucleotide Sequence
  File Name        : S_minutum_mtDNA-v1.seq
  Sequence Size    : 326535

2nd Nucleotide Sequence
  File Name        : RNA15_lcl|M76611.1[624-594]c
  Sequence Size    : 31

Query Range: 253421 - 253447
Sbjct Range: 5 - 31
Identity: 19 / 27 (70%)
Similarity: 19 / 27 (70%)
Gaps: 0 / 27 (0%)
Strand: Plus / Minus

Query  253421  TTTCTATCCATTACGACTTCCATTCTC  253447
               *******  *     ****** *****
Sbjct      31  TTTCTATGGAAACACACTTCCCTTCTC  5


1st Nucleotide Sequence
  File Name        : S_minutum_mtDNA-v1.seq
  Sequence Size    : 326535

2nd Nucleotide Sequence
  File Name        : RNA16_lcl|M76611.1[3-33]
  Sequence Size    : 31

Query Range: 51961 - 51991
Sbjct Range: 1 - 30
Identity: 21 / 31 (67%)
Similarity: 21 / 31 (67%)
Gaps: 1 / 31 (3%)
Strand: Plus / Minus

Query  51961  TATTGTGCTAGAGATAAGGGACCCAAAAGGC  51991
              ****** *** *  ** * **  * *** **
Sbjct     30  TATTGTTCTACA-TTACGAGATACCAAAAGC  1
```

```
1st Nucleotide Sequence
  File Name        : S_minutum_mtDNA-v1.seq
  Sequence Size    : 326535

2nd Nucleotide Sequence
  File Name        : RNA17_lcl|M76611.1[126-165]

Query Range: 76949 - 76985
Sbjct Range: 1 - 37
Identity: 24 / 37 (64%)
Similarity: 24 / 37 (64%)
Gaps: 0 / 37 (0%)
Strand: Plus / Plus

Query  76949  TTTTACAAGCCTTTCTAGAGATAAATGTGGACTTTTA  76985
              ****  *  **   ** *  * ******  *******
Sbjct      1  TTTTTGATCCCAGGCTGGTAAAAAATGTAAACTTTTA  37



1st Nucleotide Sequence
  File Name        : S_minutum_mtDNA-v1.seq
  Sequence Size    : 326535

2nd Nucleotide Sequence
  File Name        : RNA18_lcl|M76611.1[4964-4988]
  Sequence Size    : 25

Query Range: 300291 - 300312
Sbjct Range: 1 - 22
Identity: 17 / 22 (77%)
Similarity: 17 / 22 (77%)
Gaps: 0 / 22 (0%)
Strand: Plus / Plus

Query  300291  TGCTTTTTATTGCATGCCAGGT  300312
               ** *   *********** ***
Sbjct       1  TGTTCGGTATTGCATGCCTGGT  22


1st Nucleotide Sequence
  File Name        : S_minutum_mtDNA-v1.seq
  Sequence Size    : 326535

2nd Nucleotide Sequence
  File Name        : RNA19_lcl|M76611.1[5576-5547]c
  Sequence Size    : 30

Query Range: 308063 - 308089
Sbjct Range: 6 - 30
Identity: 21 / 27 (77%)
Similarity: 21 / 27 (77%)
Gaps: 2 / 27 (7%)
Strand: Plus / Minus

Query  308063  AATCTTATAAGCCCATGCAATCACATA  308089
               *****  ***  ****** * ******
Sbjct      30  AATCTCGTAA--CCATGCCAACACATA  6


1st Nucleotide Sequence
  File Name        : S_minutum_mtDNA-v1.seq
  Sequence Size    : 326535

2nd Nucleotide Sequence
  File Name        : RNA20_lcl|M76611.1[34-71]
  Sequence Size    : 38

Query Range: 242197 - 242225
Sbjct Range: 7 - 35
Identity: 20 / 29 (68%)
```

```
Similarity: 20 / 29 (68%)
Gaps: 0 / 29 (0%)
Strand: Plus / Plus

Query  242197  GACAAATAAATCCATATAAATAAAAGATT  242225
               ***    ****** * * * ******* *
Sbjct       7  GACCGTCAAATCCTTTTCATTAAAAGAGT  35


1st Nucleotide Sequence
  File Name        : S_minutum_mtDNA-v1.seq
  Sequence Size    : 326535

2nd Nucleotide Sequence
  File Name        : RNA21_lcl|M76611.1[1807-1829]
  Sequence Size    : 23


Query Range: 122845 - 122864
Sbjct Range: 1 - 20
Identity: 16 / 20 (80%)
Similarity: 16 / 20 (80%)
Gaps: 0 / 20 (0%)
Strand: Plus / Minus

Query  122845  CATAAACCAACAAATTAATA  122864
               * ***********  *** *
Sbjct      20  CTTAAACCAACAACATAACA  1


1st Nucleotide Sequence
  File Name        : S_minutum_mtDNA-v1.seq
  Sequence Size    : 326535

2nd Nucleotide Sequence
  File Name        : RNA22_lcl|M76611.1[5378-5341]c
  Sequence Size    : 38

Query Range: 57665 - 57699
Sbjct Range: 4 - 37
Identity: 24 / 35 (68%)
Similarity: 24 / 35 (68%)
Gaps: 1 / 35 (2%)
Strand: Plus / Plus

Query  57665  AAATACACAAAATAAATGGATTTTTATTGAAAAGA  57699
              *** ***    *** * ****** *  ****** *
Sbjct      4  AAAGACA-TCGATATACGGATTTCTCCTGAAAAAA  37


1st Nucleotide Sequence
  File Name        : S_minutum_mtDNA-v1.seq
  Sequence Size    : 326535

2nd Nucleotide Sequence
  File Name        : RNA23t_lcl|M76611.1[171-203]
  Sequence Size    : 33

Query Range: 186459 - 186487
Sbjct Range: 2 - 30
Identity: 20 / 29 (68%)
Similarity: 20 / 29 (68%)
Gaps: 0 / 29 (0%)
Strand: Plus / Minus

Query  186459  GAATTATTATTTTATTATCTGTATCTATT  186487
               ** *****    *   ****** ******
Sbjct      30  GAGTTATTGGCCTGGCATCTGTTTCTATT  2
```

```
1st Nucleotide Sequence
  File Name        : S_minutum_mtDNA-v1.seq
  Sequence Size    : 326535

2nd Nucleotide Sequence
  File Name        : RNA24t_lcl|M76611.1[262-224]c
  Sequence Size    : 39

Query Range: 23626 - 23665
Sbjct Range: 2 - 39
Identity: 27 / 40 (67%)
Similarity: 27 / 40 (67%)
Gaps: 2 / 40 (5%)
Strand: Plus / Minus

Query  23626  TTGTAAGTAAGAAAAGATTCAATTTTAGAGAGAACTTTAC  23665
              ** * ** ***  * *  * ******  * ******* **
Sbjct     39  TTTTTAGCAAG--ACGGATAAATTTTTCATAGAACTTAAC  2


1st Nucleotide Sequence
  File Name        : S_minutum_mtDNA-v1.seq
  Sequence Size    : 326535

2nd Nucleotide Sequence
  File Name        : RNA25t_lcl|M76611.1[283-262]c
  Sequence Size    : 22

Query Range: 302697 - 302717
Sbjct Range: 1 - 21
Identity: 16 / 21 (76%)
Similarity: 16 / 21 (76%)
Gaps: 0 / 21 (0%)
Strand: Plus / Plus

Query  302697  ATCTTTGGCTTTAGGATGATA  302717
               *******  *  * *******
Sbjct       1  ATCTTTGCATGGATGATGATA  21


1st Nucleotide Sequence
  File Name        : S_minutum_mtDNA-v1.seq
  Sequence Size    : 326535

2nd Nucleotide Sequence
  File Name        : RNA26t_lcl|M76611.1[1764-1806]
  Sequence Size    : 43

Query Range: 186374 - 186415
Sbjct Range: 1 - 42
Identity: 29 / 43 (67%)
Similarity: 29 / 43 (67%)
Gaps: 2 / 43 (4%)
Strand: Plus / Minus

Query  186374  TCTTGGGTCTCTTATAAATCTATTATCATTTTTG-TCTTTATG  186415
               * **  *** * *   ******** ****   ** ****** *
Sbjct      42  TTTTTAGTCCCATGCTAATCTATT-TCATAAATGATCTTTACG  1


1st Nucleotide Sequence
  File Name        : S_minutum_mtDNA-v1.seq
  Sequence Size    : 326535

2nd Nucleotide Sequence
  File Name        : RNA27t_lcl|M76611.1[4138-4085]c
  Sequence Size    : 54

Query Range: 278662 - 278712
Sbjct Range: 1 - 51
Identity: 36 / 52 (69%)
```

Similarity: 36 / 52 (69%)
Gaps: 2 / 52 (3%)
Strand: Plus / Minus

```
Query  278662  TTTTGGCTATCCTTCATCAATAAAAATGAAAGTTTTAT-GGGATTTATTAAG  278712
               *** ** ***  * ** ** ******* *   ******    ** *******
Sbjct      51  TTTAGGGTATGATACAGCATTAAAAAT-ACCCTTTTATCCAAATCTATTAAG  1
```

supplementary fig. 4

```
1st Nucleotide Sequence
  File Name        : S_minutum_mtDNA-v1.seq
  Sequence Size    : 326535

2nd Nucleotide Sequence
  File Name        : intergenic_1_lcl|M76611.1[1488-1515]
  Sequence Size    : 28

Query Range: 292924 - 292945
Sbjct Range: 2 - 22
Identity: 19 / 22 (86%)
Similarity: 19 / 22 (86%)
Gaps: 1 / 22 (4%)
Strand: Plus / Plus

Query  292924  TATAGAAAACGGTAAGATAATG  292945
               **** * ****** ********
Sbjct       2  TATA-AGAACGGTGAGATAATG  22



1st Nucleotide Sequence
  File Name        : S_minutum_mtDNA-v1.seq
  Sequence Size    : 326535

2nd Nucleotide Sequence
  File Name        : intergenic_2_lcl|M76611.1[1681-1697]
  Sequence Size    : 17

  Unit Size to Compare = 6
  Pick up Location     = 10

Query Range: 253046 - 253059
Sbjct Range: 3 - 16
Identity: 13 / 14 (92%)
Similarity: 13 / 14 (92%)
Gaps: 0 / 14 (0%)
Strand: Plus / Minus

Query  253046  AAACCCAGTATATT  253059
               * ************
Sbjct      16  ATACCCAGTATATT  3



1st Nucleotide Sequence
  File Name        : S_minutum_mtDNA-v1.seq
  Sequence Size    : 326535

2nd Nucleotide Sequence
  File Name        : intergenic_3_|M76611.1[2024-2036]
```

```
  Sequence Size    : 13

Query Range: 38496 - 38507
Sbjct Range: 1 - 12
Identity: 11 / 12 (91%)
Similarity: 11 / 12 (91%)
Gaps: 0 / 12 (0%)
Strand: Plus / Minus

Query  38496  TAACATGAGGAT  38507
              ********** *
Sbjct     12  TAACATGAGGCT  1



1st Nucleotide Sequence
  File Name        : S_minutum_mtDNA-v1.seq
  Sequence Size    : 326535

2nd Nucleotide Sequence
  File Name        : intergenic_4_lcl|M76611.1[4697-4715]
  Sequence Size    : 19

Query Range: 187217 - 187234
Sbjct Range: 2 - 19
Identity: 16 / 18 (88%)
Similarity: 16 / 18 (88%)
Gaps: 0 / 18 (0%)
Strand: Plus / Plus

Query  187217  AATATGATTGGAAATTAT  187234
               * *********** ***
Sbjct       2  ACTATGATTGGAAAATAT  19



1st Nucleotide Sequence
  File Name        : S_minutum_mtDNA-v1.seq
  Sequence Size    : 326535

2nd Nucleotide Sequence
  File Name        : intergenic_5_lcl|M76611.1[4866-4886]
  Sequence Size    : 21

  Unit Size to Compare = 6
  Pick up Location     = 10

Query Range: 117595 - 117610
Sbjct Range: 5 - 20
Identity: 15 / 16 (93%)
Similarity: 15 / 16 (93%)
Gaps: 0 / 16 (0%)
Strand: Plus / Minus
```

```
Query  117595  CTATTATAAACCAAAA  117610
               ************* **
Sbjct      20  CTATTATAAACCAGAA  5



1st Nucleotide Sequence
  File Name        : S_minutum_mtDNA-v1.seq
  Sequence Size    : 326535

2nd Nucleotide Sequence
  File Name        : intergenic_6_|M76611.1[4946-4963]
  Sequence Size    : 18

  Unit Size to Compare = 6
  Pick up Location     = 10

Query Range: 15137 - 15154
Sbjct Range: 1 - 18
Identity: 15 / 18 (83%)
Similarity: 15 / 18 (83%)
Gaps: 0 / 18 (0%)
Strand: Plus / Plus

Query  15137  TATAGACTTATCGATAGA  15154
              * ** *********** *
Sbjct      1  TTTATACTTATCGATAAA  18
```

supplementary fig. 5

```
Database:  S_minutum_mtDNA-v1.seq
           1 sequences; 326,535 total letters

Query= lcl|KF651061.1_cdsid_AHA41645.1 [gene=nad1] [protein=NADH
dehydrogenase subunit 1] [protein_id=AHA41645.1]
[location=complement(15831..16814)]

Length=984
                                                                      Score     E
Sequences producing significant alignments:                          (Bits)  Value
N

  S_minutum-mt                                                        33.6    0.005
1


> S_minutum-mt
Length=326535

 Score = 33.6 bits (67),  Expect = 0.005
 Identities = 14/54 (26%), Positives = 29/54 (54%), Gaps = 0/54 (0%)
 Frame = -2/-1

Query  836     VALIHIkk*tkkKILNPIGIINQPPKNKITVKIDINTILAYSAKKNKANIIEEY  675
               + L+H      + ++ P+G+ +    NK T+  +  T+L ++  K+K  I+E Y
Sbjct  295788  IMLVHWWSVYNEILVLPLGLTSGSSVNKYTIPENPGTLLPWAC*KSKLGILESY  295627



Query= lcl|KF651061.1_cdsid_AHA41637.1 [gene=nad2] [protein=NADH
dehydrogenase subunit 2] [protein_id=AHA41637.1]
[location=complement(577..1965)]

Length=1389
                                                                      Score     E
Sequences producing significant alignments:                          (Bits)  Value
N

  S_minutum-mt                                                        29.9    0.069
5


> S_minutum-mt
Length=326535

 Score = 29.9 bits (59),  Expect(5) = 0.069
 Identities = 10/17 (59%), Positives = 15/17 (88%), Gaps = 0/17 (0%)
 Frame = +1/+2

Query  289     LIINSFDFIVFLFSMEL  339
               LIIN+++FI+F+F M L
Sbjct  176468  LIINNYEFIIFIFCMRL  176518


 Score = 27.6 bits (54),  Expect(5) = 0.069
 Identities = 10/21 (48%), Positives = 15/21 (71%), Gaps = 0/21 (0%)
 Frame = +1/+2

Query  238     KKPVIFMIFVFIFLSFYLIIN  300
               K  +IFM F  IFLS+Y +++
Sbjct  128516  KNLIIFMFFSSIFLSYYSVLS  128578


 Score = 26.7 bits (52),  Expect(5) = 0.069
 Identities = 11/30 (37%), Positives = 16/30 (53%), Gaps = 0/30 (0%)
 Frame = +1/+1
```

```
Query  622     IWLPDIYGSLSYFIISFFSVFPKMSILFFI  711
               I+ P  YG L  FII F     + ++ F+I
Sbjct  261157  IYFPVFYGPLQEFIIVFMVQISQPTVYFYI  261246
```

```
 Score = 25.4 bits (49),  Expect(5) = 0.069
 Identities = 8/16 (50%), Positives = 12/16 (75%), Gaps = 0/16 (0%)
 Frame = +1/+3

Query  1123    LVFNSYYSFYVIFALG  1170
               L F+ YY FY++ +LG
Sbjct  282516  LSFHRYYQFYILSSLG  282563
```

```
 Score = 22.6 bits (43),  Expect(5) = 0.069
 Identities = 4/14 (29%), Positives = 9/14 (64%), Gaps = 0/14 (0%)
 Frame = +3/+3

Query  1053    YVFFNKWFSTFWWL  1094
               ++++  WF   WW+
Sbjct  261237  FLYYLLWFLCLWWI  261278
```

```
Query= lcl|KF651061.1_cdsid_AHA41661.1 [gene=orf111] [protein=orf111]
[protein_id=AHA41661.1] [location=27307..27642]

Length=336
                                                                      Score     E
Sequences producing significant alignments:                          (Bits)  Value
N

  S_minutum-mt                                                        27.6    0.084
1


> S_minutum-mt
Length=326535

 Score = 27.6 bits (54),  Expect = 0.084
 Identities = 8/16 (50%), Positives = 12/16 (75%), Gaps = 0/16 (0%)
 Frame = +1/-3

Query  187     FNRYFYIFNYDYYLKN  234
               FN ++Y+  +DYY KN
Sbjct  238129  FNLFYYVLLWDYYSKN  238082
```

```
Query= lcl|KF651061.1_cdsid_AHA41668.1 [gene=rps19] [protein=ribosomal
protein S19] [protein_id=AHA41668.1] [location=31475..31699]

Length=225
                                                                      Score     E
Sequences producing significant alignments:                          (Bits)  Value
N

  S_minutum-mt                                                        26.7    0.088
1


> S_minutum-mt
Length=326535

 Score = 26.7 bits (52),  Expect = 0.088
 Identities = 8/23 (35%), Positives = 14/23 (61%), Gaps = 0/23 (0%)
 Frame = +1/-2

Query  73      RSFPIVSYMVGKYYYIPNGKKYK  141
               R  P +S+++  Y+Y P   +YK
Sbjct  238439  RERPKLSFIISNYWYAPRKSRYK  238371
```

```
Query= lcl|AF396436.1_gene_40 [gene=cox2] [location=30802..32616]

Length=1815
                                                                      Score     E
Sequences producing significant alignments:                          (Bits)  Value
N

  S_minutum-mt                                                        30.4    0.088
1


> S_minutum-mt
Length=326535

 Score = 30.4 bits (60),  Expect = 0.088
 Identities = 13/34 (38%), Positives = 18/34 (53%), Gaps = 0/34 (0%)
 Frame = -3/-2

Query  1357    RLINMVYQYILLNQFYYHCHPYSI*FEVLQLFFY  1256
               R+I   YQ +LL  FY  CH  S    ++ L +Y
Sbjct  182834  RIIKSCYQRLLLRHFYLPCHLTSPSKTIIFLIYY  182733


Query= lcl|AF396436.1_gene_14 [gene=rps12]
[location=complement(9567..9968)]

Length=402
                                                                      Score     E
Sequences producing significant alignments:                          (Bits)  Value
N

  S_minutum-mt                                                        31.3    0.008
1


> S_minutum-mt
Length=326535

 Score = 31.3 bits (62),  Expect = 0.008
 Identities = 10/16 (63%), Positives = 13/16 (81%), Gaps = 0/16 (0%)
 Frame = -1/+3

Query  117    CNNSSFHNNALFLRIN  70
              CNNS FHNN++ + IN
Sbjct  42240  CNNSKFHNNSVIICIN  42287


Query= lcl|AF396436.1_gene_17 [gene=rps14] [location=11898..12203]

Length=306
                                                                      Score     E
Sequences producing significant alignments:                          (Bits)  Value
N

  S_minutum-mt                                                        29.5    0.021
1


> S_minutum-mt
Length=326535

 Score = 29.5 bits (58),  Expect = 0.021
 Identities = 11/32 (34%), Positives = 21/32 (66%), Gaps = 0/32 (0%)
 Frame = -3/+1

Query  280    VIDLK*PFYLIHDVLFL*TYLHFFYNH*IYIF  185
              +I LK PF +++ +LF   +LH+ Y   +++F
Sbjct  61420  IIHLKVPFSILNLILFSYLFLHYIYILKLFVF  61515


Query= lcl|AF396436.1_gene_30 [gene=ymf61]
```

```
[location=complement(19610..20326)]

Length=717
                                                                      Score     E
Sequences producing significant alignments:                          (Bits)  Value
N

  S_minutum-mt                                                        34.5    0.002
1


> S_minutum-mt
Length=326535

 Score = 34.5 bits (69),  Expect = 0.002
 Identities = 15/29 (52%), Positives = 19/29 (66%), Gaps = 0/29 (0%)
 Frame = -2/-1

Query  542     FLKYFNLIIASFLVPNLIFLFLKKFSKYS  456
               FL YF LII+S   P+L F+ LK   KY+
Sbjct  186354  FLNYFLLIISSSRTPSL*FVILKNLRKYN  186268


Query= lcl|AF396436.1_gene_25 [gene=ymf65]
[location=complement(15758..16840)]

Length=1083
                                                                      Score     E
Sequences producing significant alignments:                          (Bits)  Value
N

  S_minutum-mt                                                        29.9    0.069
1


> S_minutum-mt
Length=326535

 Score = 29.9 bits (59),  Expect = 0.069
 Identities = 11/33 (33%), Positives = 18/33 (55%), Gaps = 0/33 (0%)
 Frame = +3/+3

Query  612    SYDWFLYNIFILFNRFYRMIYYKFYSSF*QSNQ  710
              S+DW    I+  F R++ ++YY F      +NQ
Sbjct  41820  SFDWVFMLIYYRFTRWWPLLYYHFMH*IKPNNQ  41918


Query= lcl|AF396436.1_gene_53 [gene=ymf70] [location=42427..42696]

Length=270
                                                                      Score     E
Sequences producing significant alignments:                          (Bits)  Value
N

  S_minutum-mt                                                        28.6    0.033
1


> S_minutum-mt
Length=326535

 Score = 28.6 bits (56),  Expect = 0.033
 Identities = 9/21 (43%), Positives = 13/21 (62%), Gaps = 0/21 (0%)
 Frame = +1/-1

Query  187   TYFDYIYLKSIPCLFLIIFFT  249
             T+  YIYL + P   ++ FFT
Sbjct  5109  TFLSYIYLSAFPFYMIVTFFT  5047


Query= lcl|AF396436.1_gene_37 [gene=ymf77]
```

```
[location=complement(22781..26746)]

Length=3966
                                                                      Score     E
Sequences producing significant alignments:                          (Bits)  Value
N

  S_minutum-mt                                                        33.6    0.021
1


> S_minutum-mt
Length=326535

 Score = 33.6 bits (67),  Expect = 0.021
 Identities = 17/34 (50%), Positives = 20/34 (59%), Gaps = 0/34 (0%)
 Frame = -1/-3

Query  3255    YYSFLSKFCFFIILVYIFSMSFKF*SH**IFLLV  3154
               YY F+  FC   +L  IF  SF F*S   +FLLV
Sbjct  291199  YYHFIKVFCSIALLPSIF*ESFHF*SLIIVFLLV  291098


Query= lcl|AF396436.1_gene_12 [gene=ymf74]
[location=complement(8576..9049)]

Length=474
                                                                      Score     E
Sequences producing significant alignments:                          (Bits)  Value
N

  S_minutum-mt                                                        28.1    0.095
1


> S_minutum-mt
Length=326535

 Score = 28.1 bits (55),  Expect = 0.095
 Identities = 15/45 (33%), Positives = 23/45 (51%), Gaps = 0/45 (0%)
 Frame = +1/-1

Query  238     LNTLSIKNYKNKNLIISNNNQLDIIKFQKFLFIIDNKYVNSLICD  372
               L  LS K  +N N+II    +   I    F+F+  + Y++S  CD
Sbjct  105060  LGFLSYKIPRNYNIIIILFARYYSINLYLFIFLCLSVYISSTFCD  104926


Query= lcl|AF396436.1_gene_54 [gene=nad4] [location=42707..44224]

Length=1518
                                                                      Score     E
Sequences producing significant alignments:                          (Bits)  Value
N

  S_minutum-mt                                                        33.1    0.011
1


> S_minutum-mt
Length=326535

 Score = 33.1 bits (66),  Expect = 0.011
 Identities = 15/42 (36%), Positives = 22/42 (52%), Gaps = 0/42 (0%)
 Frame = +1/-3

Query  442    YIFSFTIFLLIVYVYVTVSNILLFFMCYELLLIPSFLIVYFV  567
              +I  FTIF++I Y Y    +I    +     LI S  +VY+V
Sbjct  54337  FIILFTIFIIISYFYYLFYSIFALLLIIIYYLI*SVFLVYYV  54212
```
