# Supplementary material for: The Large Mitochondrial Genome of Symbiodinium minutum Reveals Conserved Noncoding Sequences between Dinoflagellates and Apicomplexans
Source: Genome Biol Evol. 2015 Jul 20;7(8):2237–44. doi: 10.1093/gbe/evv137 (PMC4558855; doi:10.1093/gbe/evv137)
Supplement: Supplementary Data [file supp_evv137_suppl_data.zip › Supple_Figs4-7_Shoguchi.pdf]

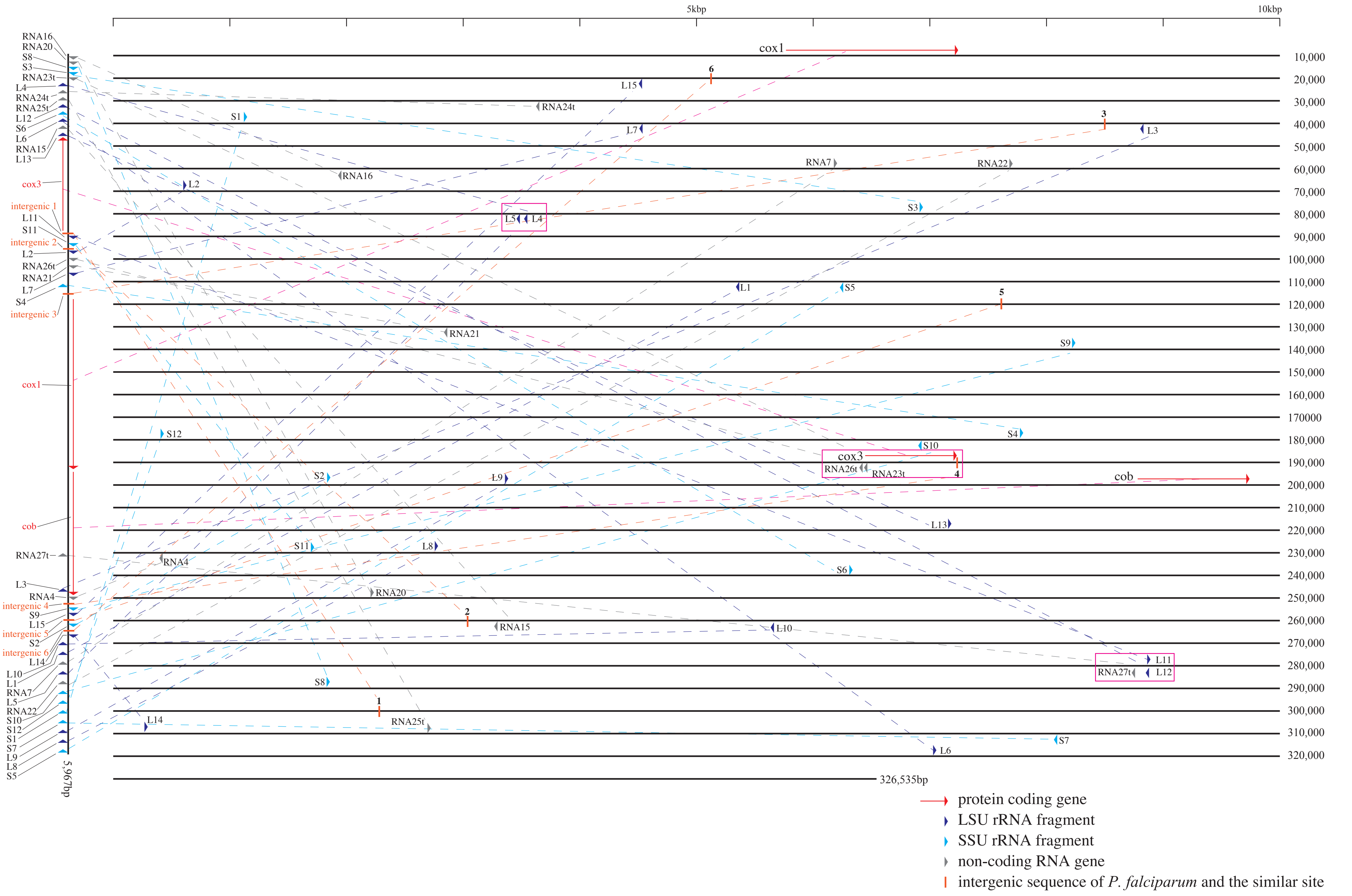
5kbp
10kbp
cox1
cox3
cob
10,000
20,000
30,000
40,000
50,000
60,000
70,000
80,000
90,000
100,000
110,000
120,000
130,000
140,000
150,000
160,000
170000
180,000
190,000
200,000
210,000
220,000
230,000
240,000
250,000
260,000
270,000
280,000
290,000
300,000
310,000
320,000
326,535bp
5,967bp
intergenic 1
intergenic 2
intergenic 3
intergenic 4
intergenic 5
intergenic 6
RNA16
RNA20
RNA23t
RNA24t
RNA25t
RNA26t
RNA21
RNA27t
RNA4
RNA15
RNA7
RNA22
L1 L2 L3 L4 L5 L6 L7 L8 L9 L10 L11 L12 L13 L14 L15
S1 S2 S3 S4 S5 S6 S7 S8 S9 S10 S11 S12
protein coding gene
LSU rRNA fragment
SSU rRNA fragment
non-coding RNA gene
intergenic sequence of P. falciparum and the similar site

supplementary fig. 5

```
1st Nucleotide Sequence
  File Name        : S_minutum_mtDNA-v1.seq
  Sequence Size    : 326535

2nd Nucleotide Sequence
  File Name        : gi|357196841|emb|HE610733.1|
  Sequence Size    : 145

Query Range: 8175 - 8308
Sbjct Range: 4 - 134
Identity: 78 / 136 (57%)
Similarity: 78 / 136 (57%)
Gaps: 7 / 136 (5%)
Strand: Plus / Minus

Query  8175  TCTAGAAGAGGAAAGGTAAATAAGAATTATTAATAAAAATAGGAATTATATGATTAAGGT  8234
             *** *** ******  ** **  * ****** *    ******  * **** * *  * *
Sbjct   134  TCTCGAA-AGGAAAAATATATTTGGATTATTGAGGCTAATAGGTGTGATAT-AGTGTGTT  77

Query  8235  AAGAGAATCCATAAGGATTAC-ACCAAAACACTTTATTATCCTCACCA-CAATATGCAGC  8292
              * *      ** *  ** ** ***** *  * ** *  *  * * ** *  **** * *
Sbjct    76  CA-ATCGCTGATCA-TATAACTACCAACATTCCTTTTACTTGTTATCATCTGTATGGA-C  20

Query  8293  ATAATAAGCCACCAAA  8308
              ***    *   ****
Sbjct    19  GTAACCTCCAGACAAA  4


1st Nucleotide Sequence
  File Name        : S_minutum_mtDNA-v1.seq
  Sequence Size    : 326535

2nd Nucleotide Sequence
  File Name        : gi|357196842|emb|HE610734.1|
  Sequence Size    : 120

Query Range: 2692 - 2788
Sbjct Range: 4 - 99
Identity: 56 / 97 (57%)
Similarity: 56 / 97 (57%)
Gaps: 1 / 97 (1%)
Strand: Plus / Minus

Query  2692  AATAATCTCAAGCATAATACATGGAATGGCCAATCATAAGTCTACCATAATTCCTTTTCT  2751
             * ***  *  *** *     *** *** **  ******   ******  *********
Sbjct    99  AGTAAGGTTGAGCGTGGATAATGCAATCGCTGATCATATAACTACCAACATTCCTTTTAC  40

Query  2752  TAGTCTCTCCAGGGTGGGGTCAGAGCTTAAAGTTGAA  2788
             * ** * **    **  ** *  * * *  **   **
Sbjct    39  TTGT-TATCATCTGTATGGACGTAACCTCCAGACAAA  4


1st Nucleotide Sequence
  File Name        : S_minutum_mtDNA-v1.seq
  Sequence Size    : 326535
```

```
2nd Nucleotide Sequence
  File Name        : gi|357196843|emb|HE610735.1|
  Sequence Size    : 61

Query Range: 237508 - 237557
Sbjct Range: 6 - 54
Identity: 31 / 50 (62%)
Similarity: 31 / 50 (62%)
Gaps: 1 / 50 (2%)
Strand: Plus / Plus

Query  237508  AATAAGAAATTATTATTAATTATCTGTGGAAACCAGTCCGGCGGCTTTAA  237557
               **  ******* *******    **  * *  ** *  *** **  * **
Sbjct       6  AAGCAGAAATTGTTATTAA-GCGCTAGGAAGTCCTGGACGGTGGTCTAAA  54


1st Nucleotide Sequence
  File Name        : S_minutum_mtDNA-v1.seq
  Sequence Size    : 326535

2nd Nucleotide Sequence
  File Name        : gi|357196844|emb|HE610736.1|
  Sequence Size    : 76

Query Range: 170019 - 170092
Sbjct Range: 5 - 76
Identity: 40 / 74 (54%)
Similarity: 40 / 74 (54%)
Gaps: 2 / 74 (2%)
Strand: Plus / Minus

Query  170019  TTTTATAGATTAATTATTGGTATTACATGGCCAACGGCCGGGTACGACAGGTTCCAGTGG  170078
               **** *   * * ***  ***    * *   * **  *  * ******     ***
Sbjct      76  TTTTTTTTTTCAGTTACAGGTTCACCGTTAACTAC--CATGTTACGACTTCGCCCATGTA  19

Query  170079  GTCTCGGTTGATAC  170092
                ***  ********
Sbjct      18  CTCTGCGTTGATAC  5


1st Nucleotide Sequence
  File Name        : S_minutum_mtDNA-v1.seq
  Sequence Size    : 326535

2nd Nucleotide Sequence
  File Name        : gi|357196837|emb|HE610729.1|
  Sequence Size    : 73

Query Range: 118217 - 118262
Sbjct Range: 11 - 54
Identity: 31 / 46 (67%)
Similarity: 31 / 46 (67%)
Gaps: 2 / 46 (4%)
Strand: Plus / Plus

Query  118217  AATAAATTATAGAATTATAAAGGCATGACAGGGTAAAAGAATCTAA  118262
               * ****  ***** **  * * *  ******  * *******  **
Sbjct      11  ATTAAACCATAGAGTT-GAGATG-GTGACAGCCTGAAAGAATTGAA  54
```

```
1st Nucleotide Sequence
  File Name        : S_minutum_mtDNA-v1.seq
  Sequence Size    : 326535

2nd Nucleotide Sequence
  File Name        : gi|357196846|emb|HE610738.1|
  Sequence Size    : 73

Query Range: 246663 - 246729
Sbjct Range: 3 - 69
Identity: 40 / 67 (59%)
Similarity: 40 / 67 (59%)
Gaps: 0 / 67 (0%)
Strand: Plus / Plus

Query  246663  AATCGTATGTTGTCTGTAAAAACCTTCATTATTTTACCATAGGATTCCTCCATTATTAAG  246722
               **   *** **   **** ********* ** * *   ******   *   *    * **
Sbjct       3  AAAGATATTTTTATTGTACAAACCTTCAATAATATGTGATAGGAAGTCGTAACAGGTCAG  62

Query  246723  GTAAAAA  246729
                 *****
Sbjct      63  CAAAAAA  69


1st Nucleotide Sequence
  File Name        : S_minutum_mtDNA-v1.seq
  Sequence Size    : 326535

2nd Nucleotide Sequence
  File Name        : gi|357196845|emb|HE610737.1|
  Sequence Size    : 101

Query Range: 127765 - 127853
Sbjct Range: 10 - 97
Identity: 63 / 89 (70%)
Similarity: 63 / 89 (70%)
Gaps: 1 / 89 (1%)
Strand: Plus / Plus

Query  127765  GGTTGATCTGGGGCTGAGTCTTAATGTCTTGAGTTACTAACCGGCTTAGAGACCAGTCCA  127824
               ******* **  * * *****  *** ******** ***** ** ***  **** *  **
Sbjct      10  GGTTGATATGAAGTTAAGTCTCTATGCCTTGAGTTTCTAACTGGATTAATGACCCGGGCA  69

Query  127825  ACCGCTGCATTCACATTCTATTCTATAAA  127853
                **   ** *** ***  ***** * ***
Sbjct      70  TCCAACGCTTTC-CATAATATTCAAAAAA  97
```

```
1st Nucleotide Sequence
  File Name        : mtDNA-1-Sym.seq
  Sequence Size    : 326535

2nd Nucleotide Sequence
  File Name        : gi|387177936|gb|JX001584.1|
  Sequence Size    : 697

  Unit Size to Compare = 6
  Pick up Location     = 3

Query Range: 104961 - 105519
Sbjct Range: 1 - 545
Identity: 295 / 566 (52%)
Similarity: 295 / 566 (52%)
Gaps: 28 / 566 (4%)
Strand: Plus / Plus

Query  104961  AAAGAAATATAAATAAATATAAATTAATAGAATAATATCTGGCAAATAAAATAATAATAA  105020
               * *  ***  * **   * *  *  * *****   ****   ** *     *   * **
Sbjct       1  AGAATAATGGACATTCCTTTGCAGGATTAGAAATCTATCGCTCACACGCTCTTGGATTAC  60

Query  105021  TATTGTA--ATTCCTTGGGATCTTGTACGAGAGGAAGC-CAAGCCG-TCTATAATGCCCA  105076
                * * **  * *   * * **     * ** * **  *  ** * *  * * * *    *
Sbjct      61  GAGTCTAGGAATTAATAGAATTAGTGATGAAAAGATCCTGAAACAGAGCGAGAGTTATAA  120

Query  105077  ATCAACATT-CATTCACTCATTCCT-TCCGGTTCCGCTATGATAGCCCAAGACACAAACA  105134
                     *** * ***** ***   * *   *    ***** ***  **   *      *
Sbjct     121  GCGTCTATTACCTTCAC-CATCAATCTTAAGAGGAGCTATAATAATCC--TATCTTCCCT  177

Query  105135  TTAATTTTAGAGTTTTAGAATGGATATGAGGGTAAAGAGCCAACCAGAATATTAATAATA  105194
               **  ** **** **** ****  ** *  *   * * *   **  *  *  ** * *
Sbjct     178  TTCTTTCTAGACTTTTGGAATTTATTTTTGAAAATAAAAAGAAAAACCAAGTTTACAGAT  237

Query  105195  AAAATATAAAATAGATTTCTTTGGAGGGAACTAGTAAGACAAATAACTTCCGAGCCAATA  105254
                ** * **  *  ***      ***  * ** ** **** * ****    *  *  * *
Sbjct     238  GAATTCTATTAATGATGGAAACGGAAAG-ACAAGAAAGA-AGATAA--GACTTGTGATTG  293

Query  105255  ATTTCAAGATTAATTCACTGTAAAATTCACTTTATAATTCCCTTTACCAACCATATAATA  105314
               *    ***** *    * **   * ***    * ***  *     * *    **  *
Sbjct     294  AAAAGAAGATCAGACTAGTG---AGTTC---CTGTAA--CGTAGAAACTCTGATGAAGAG  345

Query  105315  ACTAATCCTGTATGAAGAGTTAAGGATTTTACAGATAACCGTCTATTACAAAGAATTATT  105374
               **    *  ** **     * ** *  ** **************** ** ****** ***
Sbjct     346  ACCTCGCTAGTTTGTTTTCTAAAAG--TTAACAGATAACCGTCTATAACCAAGAATGATT  403

Query  105375  GACCATTAACTGCCAAGGAACAAATCATCCAAGAAGCATTCTATCTTTCATGGACATTAC  105434
               ******* ************* **** ***** ******** ***** **********
Sbjct     404  GACCATTGACTGCCAAGGAACTAATCTTCCAA-AAGCATTCAATCTTCCATGGACATT--  460

Query  105435  TGGTTAATATTGTCGGGTCTTACACTCATTATTCCTACCGATGGCT-CCCATCACATTAA  105493
               *  ** * * ***  * **  *    **        *  ** * ** *  *  * *  *
Sbjct     461  TCCTTCA-AGTGTTTGATCAAAACTACAACTCCAAAAGAGAAGACTACAAAGAAGAAGAC  519

Query  105494  TTTGTTTTGTCTTCCCTCTCACTTTA  105519
                 *   * * * * **   ***  * *
Sbjct     520  CGTTAATGGCCGTGCCAATCATCTGA  545
```

```
Query Range: 221453 - 222136
Sbjct Range: 3 - 656
Identity: 324 / 690 (46%)
Similarity: 324 / 690 (46%)
Gaps: 42 / 690 (6%)
Strand: Plus / Plus

Query  221453  AATAAT-AAATGTAAAATGCAGGAATTATGATTACTACTTCCCAGA-GACCCAGGATGAC  221510
               ******  *   *    ****** **** **   ***   * ** * *  *  **** **
Sbjct       3  AATAATGGACATTCCTTTGCAGG-ATTA-GAAATCTATCGCTCACACGCTCTTGGATTAC  60

Query  221511  TAGAAATAACAATAAATTTAAATATTTTAATGTGGAACCGGGGTCTCTGAGGATAGAATT  221570
                **   **  *** ***  ** **   * ***   *      *   * ** *  *** **
Sbjct      61  GAG-TCTAGGAATTAATAGAATTA--GTGATGAAAAGATCCTGAAACAGA-GCGAGAGTT  116

Query  221571  CTATAATTATAAAGTTATTTTGGTTCCTTCTACCCAAATGATCATTAATAATCAAAGAAA  221630
                 ****   *  * ***  **         *    * * ** *  ********  *
Sbjct     117  --ATAAGCGTCTA-TTACCTTCACCATCAATCTTAAGAGGAGCTATAATAATCCTATCTT  173

Query  221631  TCCAAGAAAACAGAACCAAAATAAACTCCAACTCAAAGAAACTAGCGTAATCCCTGATAC  221690
                **       *   **      **  *     * ***  **  **   ** *     ***
Sbjct     174  CCCTTTCTTTCTAGACTTTTGGAATTTATTTTTGAAAATAAAAAGAAAAACCAAGTTTAC  233

Query  221691  ACCTTATAATTATTACCTTGTAACCCTGGCTCGTGCTAGAGATTTTATTAGAAGATTTTG  221750
               *  * *    *****       *  * *         * ***   **  *   *   ***
Sbjct     234  AGATGAATTCTATTAATGATGGAAACGGAAAGACAAGAAAGAAGATAAGACTTGTGATTG  293

Query  221751  GGAATAA-TCCTGAGT-GGAAACGCCTGCTCCATCCCGAGCCTCTGATCAATCTGTATGG  221808
                 ** **   * ** * *  *   ****   * *   **  ******* **   *
Sbjct     294  AAAAGAAGATCAGACTAGTGAGTTCCTGTAACGT--AGAAACTCTGATGAA-GAGACCTC  350

Query  221809  GCAATCATCAATCATCAAATGTAAACTCTAGTAAAGCGACGGCCTAACCAAATATTAAT-  221867
               ** *   *   *  * *** ** **    **  ** ** *    *******  ** * *
Sbjct     351  GCTAGTTTGTTTTCT-AAAAGTTAA---CAGATAACCGTC--TATAACCAAGAATGATTG  404

Query  221868  AATA-TAAATTCTTAGTTCCTTACCCTCCAGATGGACCAGGTATAACCATAAAATCTAAC  221926
               *  * * * * *  **   ** * * **** * * *     * *  *    * ** *
Sbjct     405  ACCATTGACTGCCAAGGAACTAATCTTCCAAAAGCATTCAATCTTCCATGGACATTTCCT  464

Query  221927  TTATTTTATTACTTACAAGCTTAGGATAGGAGCAGAAGATATAAAAATGTATTGATTGGT  221986
               * *  *  **  * * **    *       *  ******  ** ***      **  * *
Sbjct     465  TCAAGTGTTTGATCAAAACTACAACTCCAAAAGAGAAGA-CTACAAAGAAGAAGACCGTT  523

Query  221987  TAGTGCTGTCCTCACCTGGCAAGCAAAAAAACAATTTACTTTGATGGTGACCAAATGCCA  222046
                *  ** ** *  * *     * ** ***  ***     * **   **  *  * *
Sbjct     524  AATGGCCGTGCCAATCATCTGATCAGAAATCCAA--CCATCTGGCAGTCTCTTAGT----  577

Query  222047  ATCATAAGAGGAATAATAAAAGAAATTTCAATTTTAGATTCATTTTCTACTACATCAGGT  222106
                *   ***    *  **   *****     * **  **  **       * * * *****
Sbjct     578  -TTGGAAGCCTCAAGAT-CCAGAAA-----ACTTAGGAACCA----AAGCAATAACAGGT  626

Query  222107  TCTCCAGCTACAACCGTGTAACACCTTAAA  222136
               *******  ****************** **
Sbjct     627  TCTCCAGTAACAACCGTGTAACACCTTCAA  656
```

1st Nucleotide Sequence

```
  File Name        : mtDNA-1-Sym.seq
  Sequence Size    : 326535

2nd Nucleotide Sequence
  File Name        : gi|163913843|emb|AM773793.1|
  Sequence Size    : 82

Query Range: 127774 - 127837
Sbjct Range: 1 - 64
Identity: 53 / 64 (82%)
Similarity: 53 / 64 (82%)
Gaps: 0 / 64 (0%)
Strand: Plus / Plus

Query  127774  GGGGCTGAGTCTTAATGTCTTGAGTTACTAACCGGCTTAGAGACCAGTCCAACCGCTGCA  127833
               * ************************ * ***  * ********* *  ******* ***
Sbjct       1  GTGGCTGAGTCTTAATGTCTTGAGTTTCAAACTAGTTTAGAGACCTGGACAACCGCAGCA  60

Query  127834  TTCA  127837
               * **
Sbjct      61  TACA  64



1st Nucleotide Sequence
  File Name        : mtDNA-1-Sym.seq
  Sequence Size    : 326535

2nd Nucleotide Sequence
  File Name        : gi|387177943|gb|JX001591.1|
  Sequence Size    : 898

Query Range: 193015 - 193857
Sbjct Range: 47 - 894
Identity: 436 / 869 (50%)
Similarity: 436 / 869 (50%)
Gaps: 47 / 869 (5%)
Strand: Plus / Minus

Query  193015  AGGGCCAAGATACATAAGAAGAACACAAAGAAATACAATAAAATATGAATTATTAAG-G  193073
               * * *****    * *  *    ** ***** * *** ** **      * * ***** *
Sbjct     894  AAGACCAAGCATTAGATCACTTTCATAAAGACA-ACAGATGAAGGGCTCAATCTTAAGAG  836

Query  193074  GA----TTTTAAGGGAATGACCCACCCACAAGAAGATAGAAAAGATAGAAAGATTTTGAA  193129
               **    *  ***    **       **      *  * ** *  *  * *   *    *
Sbjct     835  GAGCTATAATAATCCTATCTTAATTCCTTTTCTATGTTGAGATTAGTGTATTTTGGAAAC  776

Query  193130  GGGATTTTAGGTAGGGTGGGTCATTGGTAAGAGG--CGGTTCCTCCTTC--ATCTTCTC-  193184
                  ** ** * ** **    *  * * * * ***  *  * *  *  **   *******
Sbjct     775  TTCATCTTTGTTATGGCAAATGCTGGATTACAGGATCTTTGCACCAGTCTTTTCTTCTCA  716

Query  193185  CCCGCCACCGCAATTGGGTCCGGCTACGCCACCGCAAGAGGGTACCACT-ACGTTACCAT  193243
               **  * *   *    *  * *  **     * *   *   * ** * **  * **  ***
Sbjct     715  CCTTCTAGTCCTGGAGTTTACATCTTAATTATCTTCATCTGTTAACCCTCTCTTTTTCAT  656

Query  193244  CACGCA-AGAAATC-TATTTTATTTATAATGTG-GCGGGACGTAGCTAGGGCCCTTACAA  193300
                  * *   * *** ** *** * ***    **  *    *  ** *          **
Sbjct     655  TTGGAATTTACATCATAATTTTTCTATCGCATGTCCATTTCTGAGATTTCTTATGGCCAG  596
```

```
Query  193301  GGAATGAAGGAGAAACCGGTGAACGGGAGGA---CAGGATGTACCCGCTACCCAATTTAC  193357
                 * * **  * ** * *    * *  * **   *  * *    * * **    ****
Sbjct     595  AAACTCAATCATAAGCTGTGACAAGAAATGAGTTCTCGTTTCCACGGTTAATAGATTTCT  536

Query  193358  ACACCA-TCCTG-CACTCATATCCAACACACGGCGGCTGTAAGGTTAGGAAAGGTCCTAA  193415
                  *** ** **  * * **     **  * **     * ******   ** ********
Sbjct     535  TGGCCAGTCATGAGATTGATGGTGTACGAAAGGAAAAGGAAAGGTT---AACGGTCCTAA  479

Query  193416  GGTAGCAAAATTCCTTGACAGGTAAGTTCCGTCCAGCATGAGCGGTGTAAGGACTTCCTC  193475
               ************************************************** *********
Sbjct     478  GGTAGCAAAATTCCTTGACAGGTAAGTTCCGTCCAGCATGAGCGGTGTAACGACTTCCTC  419

Query  193476  ACTGTCACTAGCCTCGTCTCTCAGAAATTGAGTCATCCTTGATTACGAGGAAGCCAACGG  193535
               ************** ************** **     *  ** *   *    * **
Sbjct     418  ACTGTCACTAGCCTGGTCTCTCAGAAATTAAG--GAACACGAGTCATAATCTGGCATAAC  361

Query  193536  CCTGACAATAAGACCCTGAGCACCAGTTTTCTCTCTTATTTTTAATACATTTTTAGTTAT  193595
                ** ** * ***** **        * **       ***     ***   * *   *  *
Sbjct     360  TCTCACGAGAAGACACTTCTTTTTGGATTATAAAAGTATGAACCATAAGATATAGCTCTT  301

Query  193596  CTTACTGTCGTTTA---CCTTAATAATACCTTATAATATTGTACCAGAGCTTATAGACCA  193652
               *  *   * *   *   ** * * ** ***    *   *   **  *  *  *** * *
Sbjct     300  CCAAGATTGGGAAAGTGCCGTTAGAA-ACCAACGATCCTATGACACGTCCAGATA-AGCG  243

Query  193653  ATTAAACCTGGCATATCT-AACCTCAAGATTCACTAGTTATTACTAAAGGTTTCAACTTT  193711
               ** *    *    ** ** * **  **    **     *   *   ***    **  **
Sbjct     242  ATCACCAGTCCATTAACTGAGCCGTAACCCACAAACTCTGGAAGAGAAGAAAGCATATTG  183

Query  193712  ATAT---TGATTCATTCTTGGATGATCTTTCCAGCGGTTTAAAGGAGAAATAGATTGTAA  193768
                 **     **  ***** *   * ****  *  * *  *   *         *** **
Sbjct     182  GCATAGGAAATATATTCTCGTGAGGTCTTCTCTTCTGGGTCTTGTCTTTTGGAATTCTAT  123

Query  193769  GCTTATGCCAAAGAAGTCCAATAAAAATCATAAAATCTTATATTTTTTATTGTCTTCTTT  193828
                * * **     *****   ** * * ****    * ** * ** ** ** *  *****
Sbjct     122  TCATCTG-----GAAGTTGCATCAGATTCAT--TTTGTTTTCTTATTAATAG--TTCTTG  72

Query  193829  GTTGGGCATTAACCGTCAATCCATAGGAC  193857
               ***    ****  * *  ** **** * *
Sbjct      71  GTT----ATTAGTCCTTTATTCATACGTC  47

Query Range: 185750 - 186617
Sbjct Range: 11 - 855
Identity: 408 / 879 (46%)
Similarity: 408 / 879 (46%)
Gaps: 45 / 879 (5%)
Strand: Plus / Minus

Query  185750  GAAGCGGAACCCTCTTAGGTAAGCCGGTAATAAGCATACAATAAAAACACAT-TAAAAAT  185808
               **** **  *  ***** *     *  ****** * **   ***   *   * **
Sbjct     855  GAAG-GGCTCAATCTTAAGAGGAGCTATAATAATCCTATCTTAATTCCTTTTCTATGTTG  797

Query  185809  ACAATTCTATCTAGCGGTATAGTGA-GGAAGAGATTATAAAAGTTGAGGGTTAGTGGA-A  185866
               * * *  * * *   ** *   * *     *  **   *** * *  ** ***  ***
Sbjct     796  AGATTAGTGTATTTTGGAAACTTCATCTTTGTTATGGCAAATGCT--GGATTACAGGATC  739

Query  185867  GATACTAAAGTTAATTTTATTGACATCATATCCTACGCAATACCAACCAACTACAAATCA  185926
                 * *   ***   ** *  * ** *  **  *   *   *  ** *  * *     ***
Sbjct     738  TTTGCACCAGTCTTTTCTTCTCACCTTCTAGTCCTGGAGTTTACATCTTAATTATCTTCA  679
```

```
Query  185927  TTCATTAATAAT-TCCTTGGCTAACGGTATCTAGGTTCATCCTTCCACTAACCGATGCCA  185985
               *   ****   * ** **  *    ** ** **   ****  **   *** *  *** *
Sbjct     678  TCTGTTAACCCTCTCTTTTTCATTTGGAATTTA-CATCATAATTTTTCTATCGCATGTCC  620

Query  185986  ACTATTGATTATTACTCGTTCAACCTACTGATTCCGCCAGGAGGTGAGACCTCTTAGATA  186045
               * *  ***   **  *    * *   *** * *     * *  **** *     *   *
Sbjct     619  ATTTCTGAGATTTCTTATGGCCAGAAACTCAAT--CATAAGCTGTGACAAGAAATGAGTT  562

Query  186046  AAGCTGGACACTAGTAA-CCAGATCAATCCTAATGAACCCACTGTAACCATTATAATTCC  186104
                   *   ***   ***   *  **    * * * *    * ** *     **  *
Sbjct     561  CTCGTTTCCACGGTTAATAGATTTCTTGGCCAGTCATGAGATTG-ATGGTGTACGAAAGG  503

Query  186105  GTGTCAATAGCCTTACCAACATAGGATTCTAAGATTCATTGTAAGCTTTTGGATTCATGT  186164
                     * **  * **   * ** * *   ** **** ***  **         * *  *
Sbjct     502  AAAAGGAAAGGTTAACGGTCCTAAGGTAGCAAAATTCCTTGACAG--------GTAAGTT  451

Query  186165  TCTCCCTTCTCGCTAGGTAAATCTAGAGTTCTTACACCTTAAAATTCTGGAAATATACTG  186224
                *  **  *  *   ***  * *  ** *** * ***  * *    *  *   * * * *
Sbjct     450  CCGTCCAGCATGAGCGGTGTAAC--GACTTCCT-CACTGTCACTAGCCTGGTCTCT-CAG  395

Query  186225  TCGGATACCAAGAAAATCTCTTATTTTTCTTGTTTTTTAGGGTATTATATTTC--CTGAG  186282
                     *  ** *  *    * ** *  * *   * * * *  *  * * ***   *  *
Sbjct     394  AAATTAAGGAACACGAGTCATAATCTGGCATAACTCTCACGAGAAGACACTTCTTTTTGG  335

Query  186283  ATT-TTTAAGGATAACAAACTAGAGAGATGGAGTGCGTGAGGAAGAGATTATTAGTAAGA  186341
               *** *  *** ** *   *  *** * *     * *  **   ** * *     **   *
Sbjct     334  ATTATAAAAGTATGAACCATAAGATATAGCTCTTCCAAGATTGGGAAAGTGCCGTTAGAA  275

Query  186342  AATAATTAAGAAATTTTATGTTAATATTA-TTCTCTTGGGTCTCTT-ATAAATCTATTAT  186399
               *  **  *    **   * **  * ** *    **    ***  ** *   * *  * *
Sbjct     274  ACCAACGATCCTATGACACGTCCAGATAAGCGATCACCAGTCCATTAACTGAGCCGTAAC  215

Query  186400  CATTTTTGTCTTTATGTTAATAATGCTTTCTATAAAACTCTTCACTATTTATTAATACAG  186459
               *       ***  * *  ** ** **    ***     *    * **  ****  *   *
Sbjct     214  CCACAAACTCTGGAAGAGAAGAAAGC---ATATTGGCATAGGAAATA--TATT-CTCGTG  161

Query  186460  AATTATTATTTTATTATCTGTATCTATTGGTATTTTGGAAATCATTACATTTAGAAGTTA  186519
               *  *      ** *  ****  **   * ** ******** **  * *** * ******
Sbjct     160  AGGT-----CTTCTCTTCTGGGTC---TTGTCTTTTGGAATTCTATTCATCTGGAAGTTG  109

Query  186520  CATCAGATTTATTTTGTTTTCTTATTAATACTCACCATTTATTAAT-CTTTGCTCTTATT  186578
               ********* ******************** *      ****** * ****  ** **
Sbjct     108  CATCAGATTCATTTTGTTTTCTTATTAATAGTTCTTGGTTATTAGTCCTTTATTCATA-C  50

Query  186579  ATAATTCTATTTATCTTTACAGGATTTAATATTTATTGT  186617
                *  *  *  * *****  *     * *   *** ** *
Sbjct      49  GTCTTCTTCATAATCTTCTCTTTCCTAATGCTTTCTTAT  11
```

1st Nucleotide Sequence
File Name : mtDNA-1-Sym.seq
Sequence Size : 326535

2nd Nucleotide Sequence
File Name : gi|387177950|gb|JX001598.1|
Sequence Size : 453

Query Range: 222480 - 222830
Sbjct Range: 72 - 428

Identity: 179 / 362 (49%)
Similarity: 179 / 362 (49%)
Gaps: 16 / 362 (4%)
Strand: Plus / Minus

```
Query  222480  ACCCCCCAGTTCTTT-CCAGCGGTTACCAATGATTTCCTCTTCCATATCCTAC-AGT-CA  222536
               ***  **** ***** *** ******* ** *************** ** * * * * *
Sbjct     428  ACCAGCCAGGTCTTTACCAACGGTTACAAACGATTTCCTCTTCCATTTCATTCTATTCCT  369

Query  222537  ACATATTATCCGAGTACTAGCCAGGAAATAGAAAAC----CAGATAATAATTCATTAAAT  222592
                ** *  ***    *  * *        ********    *    ***  ** ** **
Sbjct     368  GCAAAGGATCTTCTTCTTTGTAGACTTTTAGAAAACATTTCTTGAAATGGTT-ATGAACG  310

Query  222593  TCCAAGATTCATTGATAACAGCCAGTACGCGGGAAGTCTTCTGGAGATAAAACTGTATTA  222652
               *  *  * **   ***    * *  *  *     * * **    * * ***     *  *
Sbjct     309  TTTACAACTCTCGGATTCACGACTTTTGGAAAATATTTTTGAAAATAAAAGAAAAACCA  250

Query  222653  TTTTCTAAGGAATATTATCAGGATTTTTGTGTCAATCATATAGGCTGTACCTAGGA-ATC  222711
                 **   **    *** *    **  * *      *  * **  *   *** * ** ***
Sbjct     249  AGTTTACAGATGAATTCTATTAATGATGGAAAAGGTGCTCTAATCCAGACCAACGATATC  190

Query  222712  C--TGGAAATTCTTTGATTATATCCTCCGGTCGGTTGAAAAGTCATGAGATTGGAAGCGT  222769
               *  *   **   *  ***** **  **      ** *  ***  * **  *  *    *
Sbjct     189  CTCTTCCAAGCATGGGATTACATTATCTTTGATGTCGCTAAGCGAGGATCTGTGCCAGGA  130

Query  222770  CTGTAAGGTTGGGACACAAGACCCTGAGAATTTGGATGAAGAAGTCAAGCCT-TTGGTAA  222828
               ** *       * *   * *      ****  ****  **  **  ** ***   *****
Sbjct     129  CT-TCCCAAGAGCATGAACGCTTTCTAGAA-ATGGA--AAAGAGAGAATCCTAAAGGTAA  74

Query  222829  CA  222830
               **
Sbjct      73  CA  72
```

supplementary fig. 6

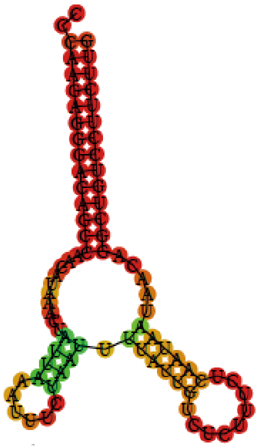

S. minutum mt 9050-8970
(similar to MIR390)

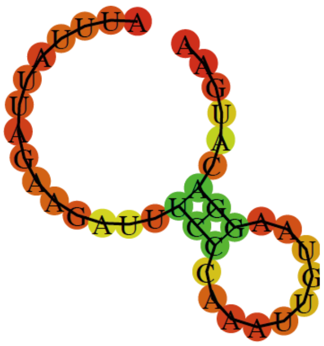

S. minutum mt 37819-37783
(similar to DGCR5)

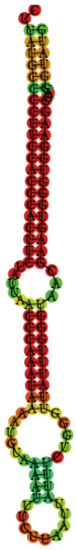

S. minutum mt
71642-71540
(similar to mir-787)

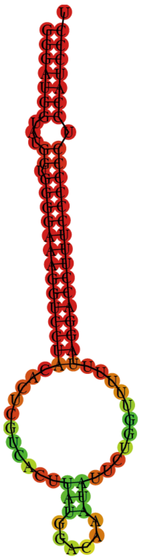

S. minutum mt
71550-71465
(similar to mir-253)

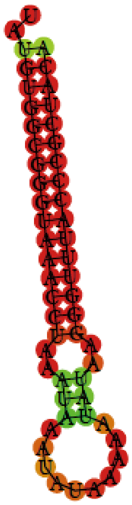

S. minutum mt
88879-88826
(similar to rox2)

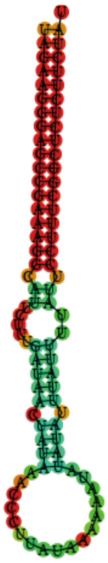

S. minutum mt
128272-128358
(similar to mir-393)

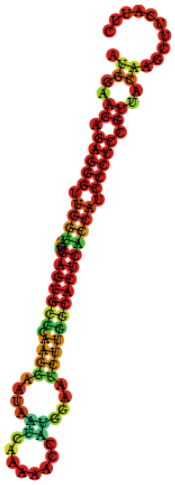

S. minutum mt 132407-132317
(similar to mir328)

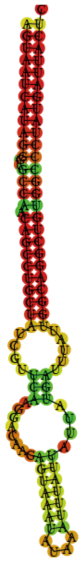

S. minutum mt
155939-156039
(similar to MIR477)

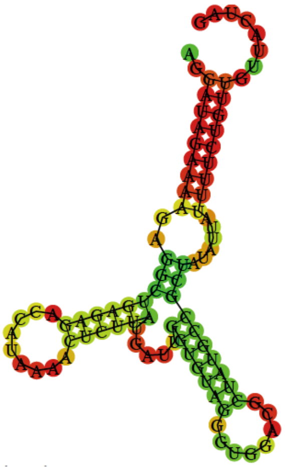

S. minutum mt 169111-169200
(similar to Virus_CITE_4)

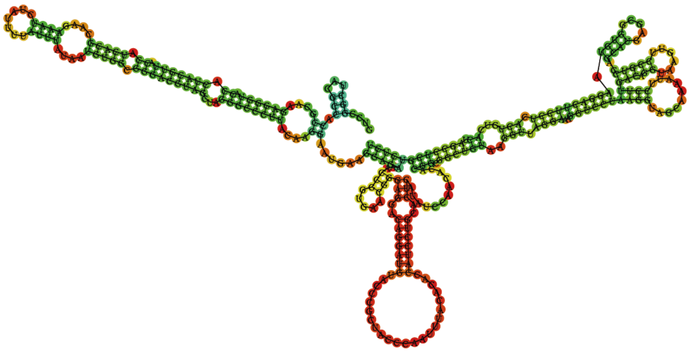

S. minutum mt 193202-193496 (similar to LSU_rRNA_bacteria)

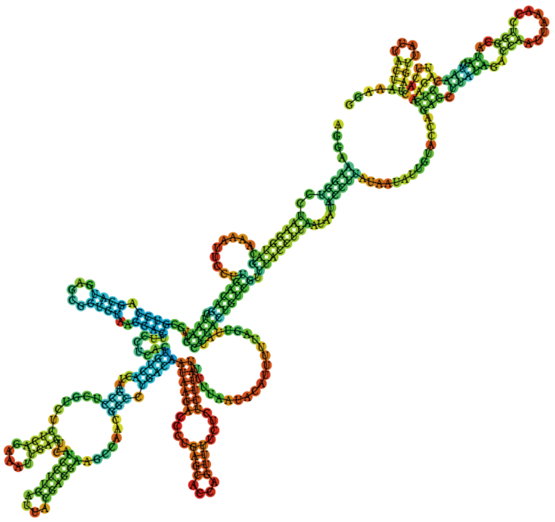

S. minutum mt 193402-193701 (similar to LSU_rRNA_archaea)

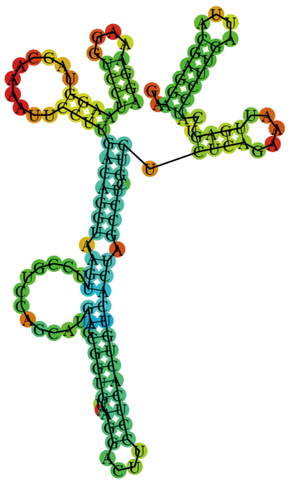

S. minutum mt 193402-193528 (similar to LSU_rRNA_bacteria)

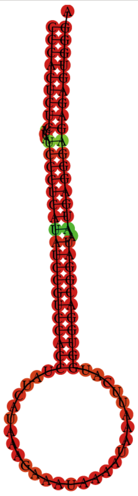

S. minutum mt 209995-210082 (similar to MIR477)

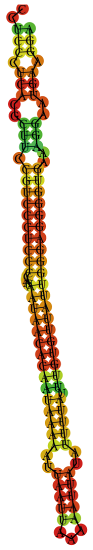

S. minutum mt 216684-216584 (similar to mir253)

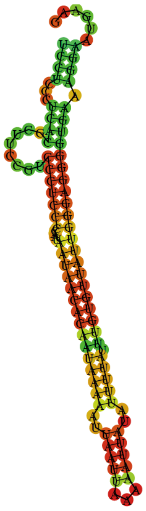

S. minutum mt 216685-216586 (similar to mir253)

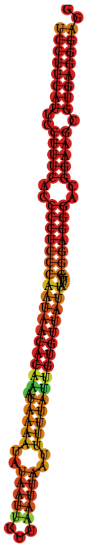

S. minutum mt 216584-216684 (similar to mir785)

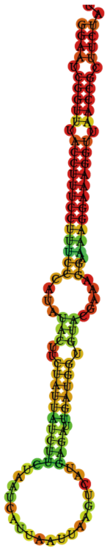

S. minutum mt 217151-217260 (similar to mir299)

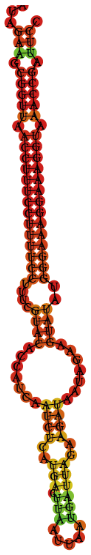

S. minutum mt 217260-217151 (similar to mir299)

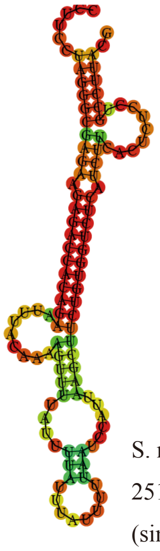

S. minutum mt 251039-251143 (similar to MIR398)

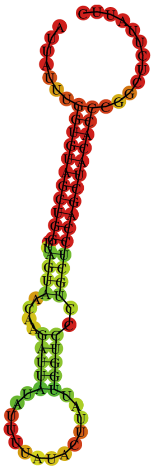

S. minutum mt 269898-269978 (similar to mir133)

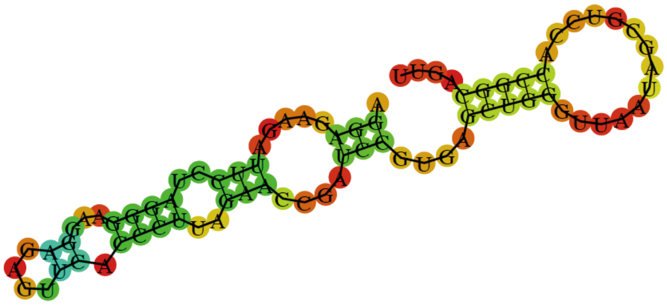

S. minutum mt 278901-278824 (similar to LSU_rRNA_bacteria)

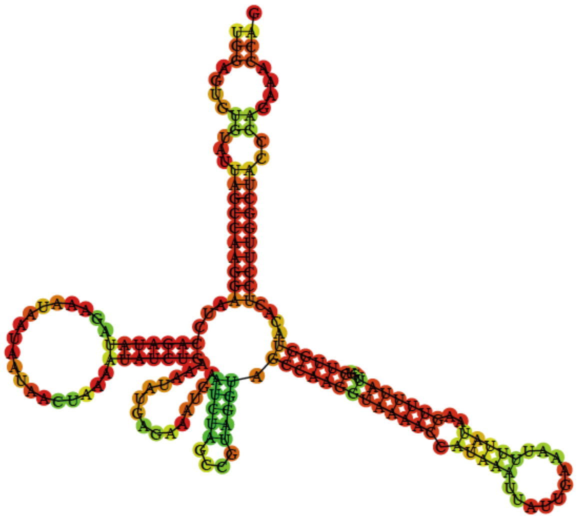

S. minutum mt 301996-301831 (similar to MIR169_2)

supplementary fig. 7

```
1st Nucleotide Sequence
  File Name        : S_minutum_mtDNA-v1.seq
  Sequence Size    : 326535

2nd Nucleotide Sequence
  File Name        : intergenic_1_lcl|M76611.1[1488-1515]
  Sequence Size    : 28

Query Range: 292924 - 292945
Sbjct Range: 2 - 22
Identity: 19 / 22 (86%)
Similarity: 19 / 22 (86%)
Gaps: 1 / 22 (4%)
Strand: Plus / Plus

Query  292924  TATAGAAAACGGTAAGATAATG  292945
               **** * ****** ********
Sbjct       2  TATA-AGAACGGTGAGATAATG  22



1st Nucleotide Sequence
  File Name        : S_minutum_mtDNA-v1.seq
  Sequence Size    : 326535

2nd Nucleotide Sequence
  File Name        : intergenic_2_lcl|M76611.1[1681-1697]
  Sequence Size    : 17

  Unit Size to Compare = 6
  Pick up Location     = 10

Query Range: 253046 - 253059
Sbjct Range: 3 - 16
Identity: 13 / 14 (92%)
Similarity: 13 / 14 (92%)
Gaps: 0 / 14 (0%)
Strand: Plus / Minus

Query  253046  AAACCCAGTATATT  253059
               * ************
Sbjct      16  ATACCCAGTATATT  3



1st Nucleotide Sequence
  File Name        : S_minutum_mtDNA-v1.seq
  Sequence Size    : 326535

2nd Nucleotide Sequence
  File Name        : intergenic_3_|M76611.1[2024-2036]
```

```
  Sequence Size    : 13

Query Range: 38496 - 38507
Sbjct Range: 1 - 12
Identity: 11 / 12 (91%)
Similarity: 11 / 12 (91%)
Gaps: 0 / 12 (0%)
Strand: Plus / Minus

Query  38496  TAACATGAGGAT  38507
              ********** *
Sbjct     12  TAACATGAGGCT  1



1st Nucleotide Sequence
  File Name        : S_minutum_mtDNA-v1.seq
  Sequence Size    : 326535

2nd Nucleotide Sequence
  File Name        : intergenic_4_lcl|M76611.1[4697-4715]
  Sequence Size    : 19

Query Range: 187217 - 187234
Sbjct Range: 2 - 19
Identity: 16 / 18 (88%)
Similarity: 16 / 18 (88%)
Gaps: 0 / 18 (0%)
Strand: Plus / Plus

Query  187217  AATATGATTGGAAATTAT  187234
               * *********** ***
Sbjct       2  ACTATGATTGGAAAATAT  19



1st Nucleotide Sequence
  File Name        : S_minutum_mtDNA-v1.seq
  Sequence Size    : 326535

2nd Nucleotide Sequence
  File Name        : intergenic_5_lcl|M76611.1[4866-4886]
  Sequence Size    : 21

  Unit Size to Compare = 6
  Pick up Location     = 10

Query Range: 117595 - 117610
Sbjct Range: 5 - 20
Identity: 15 / 16 (93%)
Similarity: 15 / 16 (93%)
Gaps: 0 / 16 (0%)
Strand: Plus / Minus
```

```
Query  117595  CTATTATAAACCAAAA  117610
               ************* **
Sbjct      20  CTATTATAAACCAGAA  5
```

```
1st Nucleotide Sequence
  File Name        : S_minutum_mtDNA-v1.seq
  Sequence Size    : 326535

2nd Nucleotide Sequence
  File Name        : intergenic_6_|M76611.1[4946-4963]
  Sequence Size    : 18

  Unit Size to Compare = 6
  Pick up Location     = 10

Query Range: 15137 - 15154
Sbjct Range: 1 - 18
Identity: 15 / 18 (83%)
Similarity: 15 / 18 (83%)
Gaps: 0 / 18 (0%)
Strand: Plus / Plus

Query  15137  TATAGACTTATCGATAGA  15154
              * ** *********** *
Sbjct      1  TTTATACTTATCGATAAA  18
```
