# Supplementary material for: The Large Mitochondrial Genome of Symbiodinium minutum Reveals Conserved Noncoding Sequences between Dinoflagellates and Apicomplexans
Source: Genome Biol Evol. 2015 Jul 20;7(8):2237–44. doi: 10.1093/gbe/evv137 (PMC4558855; doi:10.1093/gbe/evv137)
Supplement: Supplementary Data [file supp_evv137_suppl_data.zip › New_Microsoft_Office_Word_Document2.docx]

**Supplementary figures:**

**Supplementary fig. 1**

**Distributions of amino acid substitutions mediated by RNA editing in *Symbiodinium minutum* mitochondria**

High frequency of valine (V), which is mediated by RNA editing, is seen in mt proteins, suggesting conservation of hydrophobic character in dinoflagellate mitochondrial proteins (Lin et al. 2008). Lower case letters show amino acid substitutions resulting from editing. Conserved regions found by pfam domain search are surrounded by color lines. The increased frequency of valine in cox protein may be the signature of dinoflagellate RNA editing.

**Supplementary fig. 2**

**Presence or absence of conventional start or stop codons in mRNAs of *cox1*, *cob* and *cox3* predicted from protein alignments**

Red highlighting of *S. minutum* proteins indicates start M (AUG) and stop codon signals (.), showing conserved conventional start or stop codons. I (AUU, AUA) of cox1 and cox3 proteins o*f S. minutum* are also marked as candidates for start codons since they are reported as such in ciliates and apicomplexans (Feagin 1992; Edqvist et al. 2000). Identical and similar residues are marked below with “*” and “:”. “nnnnnn” shows abbreviated regions of amino acid sequences. Blue colored “Ks” represent the conceptual translation of 3’ oligo-A mRNA tails (Jackson et al. 2012). These show that the only stop codon not predicted is that of *cox1* mRNA. Predicted sequences from the NCBI database (http://www.ncbi.nlm.nih.gov/gquery/) of nucleotide and genes for the following species are found in Jackson et al. (2012). Acat, *Alexandrium catenella*; Hem, *Hermatodinium* sp.; Kmic, *Karlodinium micrum*; Ppis, *Pfiesteria piscicida*; Smi, *Symbiodinium minutum*; Symsp., *Symbiodinium* sp.

**Supplementary fig. 3**

**Gene sequence similarities between *S. minutum* and *P. falciparum***

1. The most similar regions of the *S. minutum* genome to 39 *P. falciparum* genes are

surveyed and are aligned. These support the gene map of the *S. minutum* mt genome shown in fig. 2 and supplementary fig.4. (B) Predicted secondary structures for aligned sequences of *S. minutum* and *P. falciparum* RNA genes using RNAfold (Zuker and Stiegler, 1981, <http://rna.tbi.univie.ac.at/cgi-bin/RNAfold.cgi>). (C, D) Secondary structures of L4 and L5 predicted by RNAfold (C) and by CentroidHold (D).

**Supplementary fig. 4**

**Mitochondrial gene order comparisons between *S. minutum* and *P. falciparum* with a detailed gene map.**

Genes from the *P. falciparum* mt genome (~6kbp) (left) are joined to those of *S. minutum* by colored lines (red, blue, light blue, orange and gray). Arrows and arrowheads indicate predicted gene directions. Three gene clusters, located in close proximity in *S. minutum*, are shown in pink boxes.

**Supplementary fig. 5**

**Similarities between the *S. minutum* mt genome and other dinoflagellate mt sequences**

Some regions of the *S. minutum* genome that are similar to other dinoflagellate mt sequences are shown. Details of surveyed sequences are found in the Materials and Methods.

**Supplementary fig. 6**

**Predicted secondary structures of *S. minutum* mt DNA sequences**

Secondary structures for *S. minutum* DNA sequences aligned with the Rfam family are predicted using RNAfold (<http://rna.tbi.univie.ac.at/cgi-bin/RNAfold.cgi>). Sequence locations are also shown in supplementary table 1. The presence of stem-loop structures was confirmed.

**Supplementary fig. 7**

**Unknown non-coding sequences conserved between mitochondrial genomes of *S. minutum* and *P. falciparum***

The most similar regions of the *S. minutum* genome to *P. falciparum* intergenic sequences are surveyed and are aligned. These show the sequence conservation among myzozoans (apicomplexans and dinoflagellates).
