# Supplementary material for: The Large Mitochondrial Genome of Symbiodinium minutum Reveals Conserved Noncoding Sequences between Dinoflagellates and Apicomplexans
Source: Genome Biol Evol. 2015 Jul 20;7(8):2237–44. doi: 10.1093/gbe/evv137 (PMC4558855; doi:10.1093/gbe/evv137)
Supplement: Supplementary Data [file supp_evv137_suppl_data.zip › Shoguchi_supple_table_1.pdf]

supplementary table 1. Rfam family-like sites on *S. minutum* mt DNA

Query: location of *S. minutum* genome (bp)	aligned location (bp)	aligned length (bp)	Hit by infernal search (Rfam family, description, E-value)			minimum free energy on secondary structure pediction by RNAfold (kcal/mol)
8801-9100	9050-8970	81	MIR390 (RF00689)	microRNA MIR390	0.00056	-24.4
37601-37900	37819-37783	37	DGCR5 (RF02098)	DiGeorge syndrome critical region gene 5 conserved region	0.00055	-0.4
71401-71700	71642-71540	103	mir-787 (RF00896)	microRNA mir-787	0.00095	-43.4
71401-71700	71550-71465	86	mir-253 (RF01064)	microRNA mir-253	0.00047	-42.3
88601-88900	88879-88826	54	rox2 (RF01666)	Drosophila rox2 ncRNA	0.00099	-26.3
128201-128500	128272-128358	87	mir-393 (RF02516)	mir-393 microRNA precursor family	0.00031	-31.1
132201-132500	132407-132317	91	mir-328 (RF00772)	microRNA mir-328	0.00061	-29.9
155801-156100	155939-156039	101	MIR477 (RF00780)	microRNA MIR477	0.00062	-42.2
169001-169300	169111-169200	90	Virus_CITE_4 (RF02459)	Necrovirus cap-independent translation element	0.00042	-18.8
193201-193500	193202-193496	295	LSU_rRNA_bacteria (RF02541)	Bacterial large subunit ribosomal RNA	5.10E-10	-80.6
193401-193700	193402-193701	300	LSU_rRNA_archaea (RF02540)	Archaeal large subunit ribosomal RNA	3.30E-08	-51.1
193401-193700	193402-193528	127	LSU_rRNA_bacteria (RF02541)	Bacterial large subunit ribosomal RNA	3.80E-16	-28.2
209801-210100	209995-210082	88	MIR477 (RF00780)	microRNA MIR477	1.60E-05	-50.8
216401-216700	216684-216584	101	mir-253 (RF01064)	microRNA mir-253	0.00054	-34
216401-216700	216685-216586	100	mir-253 (RF01064)	microRNA mir-253	6.50E-06	-31
216401-216700	216584-216684	101	mir-785 (RF02244)	microRNA mir-785	0.00048	-42.6
217001-217300	217151-217260	110	mir-299 (RF00756)	microRNA mir-299	5.20E-05	-33.1
217001-217300	217260-217151	110	mir-299 (RF00756)	microRNA mir-299	0.00012	-34.1
251001-251300	251039-251143	105	MIR398 (RF00695)	microRNA MIR398	0.00038	-27.8
269801-270100	269898-269978	81	mir-133 (RF00446)	mir-133 microRNA precursor family	0.00014	-22.9
278601-278900	278901-278824	78	LSU_rRNA_bacteria (RF02541)	Bacterial large subunit ribosomal RNA	4.90E-05	-16.9
301801-302100	301996-301831	166	MIR169_2 (RF00645)	microRNA MIR169_2	1.60E-05	-36.6
